# Lipogenesis mediated by OGR1 regulates metabolic adaptation to acid stress in cancer cells via autophagy

**DOI:** 10.1016/j.celrep.2022.110796

**Published:** 2022-05-10

**Authors:** Smitha Pillai, Iqbal Mahmud, Rohit Mahar, Crystal Griffith, Michael Langsen, Jonathan Nguyen, Jonathan W. Wojtkowiak, Pawel Swietach, Robert A. Gatenby, Marilyn M. Bui, Matthew E. Merritt, Patricia McDonald, Timothy J. Garrett, Robert J. Gillies

**Affiliations:** 1 Department of Cancer Physiology, H. Lee Moffitt Cancer Center and Research Institute, Tampa, FL, USA; 2 Analytical Microscopy Core, H. Lee Moffitt Cancer Center and Research Institute, Tampa, FL, USA; 3 Department of Pathology, Moffitt Cancer Center and Research Institute, Tampa, FL, USA; 4 Department of Physiology, Anatomy and Genetics Parks Road, Oxford OX1 3PT, UK; 5 Department of Radiology, H. Lee Moffitt Cancer Center and Research Institute, Tampa, FL, USA; 6 Department of Pathology, Immunology and Laboratory Medicine, University of Florida, Gainesville, FL, USA; 7 Department of Biochemistry and Molecular Biology, University of Florida, Gainesville, FL, USA; 8 Lead contact

## Abstract

Malignant tumors exhibit altered metabolism resulting in a highly acidic extracellular microenvironment. Here, we show that cytoplasmic lipid droplet (LD) accumulation, indicative of a lipogenic phenotype, is a cellular adaption to extracellular acidity. LD marker PLIN2 is strongly associated with poor overall survival in breast cancer patients. Acid-induced LD accumulation is triggered by activation of the acid-sensing G-protein-coupled receptor (GPCR) OGR1, which is expressed highly in breast tumors. OGR1 depletion inhibits acid-induced lipid accumulation, while activation by a synthetic agonist triggers LD formation. Inhibition of OGR1 downstream signaling abrogates the lipogenic phenotype, which can be rescued with OGR1 ectopic expression. OGR1-depleted cells show growth inhibition under acidic growth conditions *in vitro* and tumor formation *in vivo*. Isotope tracing shows that the source of lipid precursors is primarily autophagy-derived ketogenic amino acids. OGR1-depleted cells are defective in endoplasmic reticulum stress response and autophagy and hence fail to accumulate LDs affecting survival under acidic stress.

## In brief

Acid sensing is essential for cell survival under acidic growth conditions. Pillai et al. report that in response to extracellular acidosis, cells accumulate lipid droplets that are derived from autophagic breakdown of amino acids. This adaptive mechanism is triggered by the acid-sensing receptor OGR1 in breast cancer cells.

## Graphical Abstract

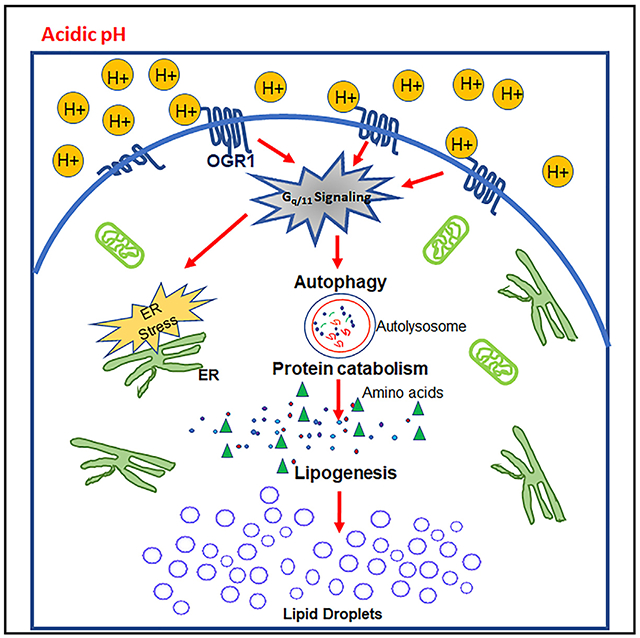

## INTRODUCTION

It is well accepted that malignant tumors metabolize significantly more glucose than surrounding normal tissue. Elevated glucose fermentation coupled with poor vascular perfusion leads to a highly acidic microenvironment, with extracellular pH values as low as 6.5 (Hashim et al., 2011). It is axiomatic that adaptations to this profoundly acidic microenvironment are necessary for tumor cells to survive and to transition from an avascular pre-invasive tumor to a malignant invasive phenotype (Gillies et al., 2008; Gatenby and Gillies, 2004, 2007; Gillies and Gatenby, 2007). One of the major survival mechanisms adopted by tumor cells under low pH is chronic activation of autophagy, the inhibition of which is selectively toxic to cells under acidic conditions (Wojtkowiak et al., 2012; Wojtkowiak and Gillies, 2012). In addition, cells that are adapted to grow under chronic acidity showed redistribution of lysosomal proteins such as LAMP-2 to the cell surface, protecting the plasma membrane from acid-mediated cytotoxicity (Damaghi et al., 2015; Glunde et al., 2003; Steffan et al., 2009, 2010). This phenotype has been localized to the invading edge of breast cancer tumors (Ji et al., 2019; Rozhin et al., 1994) and is correlated with grade in patient samples. Acid-induced lysosomal redistribution also physically separates the mammalian target of rapamycin complex 1 (mTORC1; master regulator of cellular growth and metabolism) from its regulator, small GTPase RHEB, leading to disruption of carbon metabolism and the circadian clock (Walton et al., 2018).

An additional, related adaptation to acidosis is the robust accumulation of intracellular lipid droplets (LDs), or adiposomes. These are dynamic organelles with cores of cholesterol esters (CEs) and triglycerides (TGs), surrounded by a shell comprised of polar lipids and perilipin proteins, e.g., PLIN2 (Bickel et al., 2009; Sztalryd and Kimmel, 2014). LDs function as crucial metabolic hubs by playing central roles in energy and membrane metabolism and in the production of signaling molecules (Hashemi and Goodman, 2015; Guo et al., 2009; Thiam et al., 2013). Excessive lipid storage in LDs is commonly observed in the pathogenesis of many metabolic diseases such as diabetes, obesity, fatty liver disease, atherosclerosis, and cancer (Welte, 2015; Cohen et al., 2011; Greenberg et al., 2011; Gross and Silver, 2014). Lipid storage in LDs has been suggested to be protective against nutrient starvation (Rambold et al., 2015). Studies using nuclear magnetic resonance (NMR) confirmed a subcellular origin of NMR-visible mobile lipids (MLs) (Barba et al., 1999; Delikatny et al., 2011; Boren and Brindle, 2012). Recent studies indicate that acidosis can induce the simultaneous synthesis and breakdown of fatty acids (FAs) and that this can be correlated to the reprogramming of FA metabolism through non-enzymatic acetyl coenzyme A (AcCoA)-mediated changes in mitochondrial and histone acetylation (Corbet et al., 2014, 2016; Corbet and Feron, 2017).

Here, we investigate the mechanisms regulating lipid accumulation and their role in survival under acid stress. Ion-substitution experiments show that LD accumulation is triggered by acidification of extracellular, and not intracellular, pH. Recent studies have identified a number of structurally related G-protein-coupled receptors (GPCRs) as proton-sensing receptors (e.g., TDAG8 [GPR65], OGR1 [GPR68], GPR4 [GPR19], G2A [GPR132]) that recognize extracellular pH through histidine residues located in their extracellular loops (Justus et al., 2013; Ludwig et al., 2003; Murakami et al., 2004; Seuwen et al., 2006; Wang et al., 2004; Insel et al., 2020). Herein, we show that OGR1 and TDAG8 are highly expressed in breast cancers, that OGR1 is the major receptor involved in acid-induced LD accumulation in MCF7 and T47D cells, and that OGR1 levels are related to disease progression. Activation of OGR1 induces LDs via the activation of downstream signaling through phospholipase C and pAkt pathways. CRISPR-Cas9 knockout of OGR1 (but not TDAG8) abrogated acid-induced LD accumulation and autophagy, which was rescued with ectopic expression of OGR1. We used stable isotope labeling and mass spectrometry to identify autophagically derived ketogenic amino acids as the major source of carbons for the generation of LDs. We further show that targeting LD formation is selectively lethal under acidic conditions, which are commonly observed in tumors *in vivo*.

## RESULTS

### An acidic pH induces lipid phenotype in breast cancer cell lines

**Figure 1.**
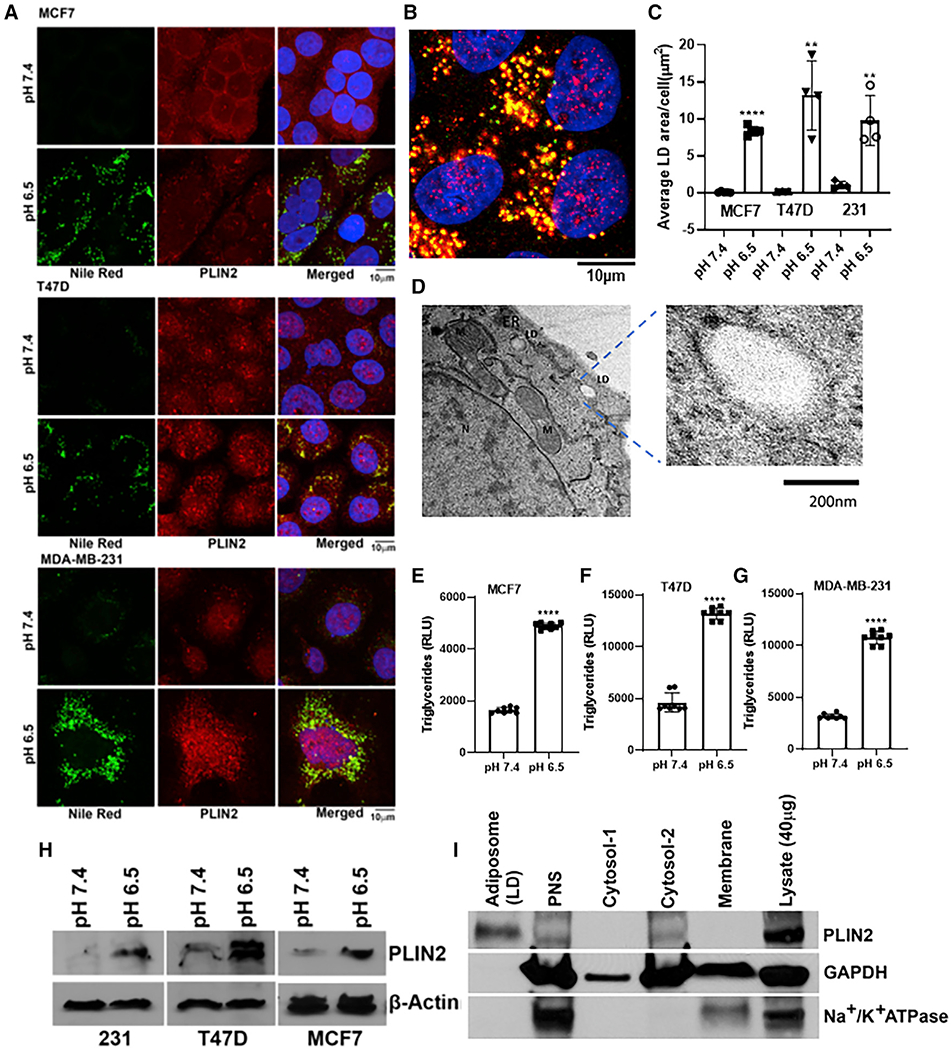
Induction of lipid phenotype by acidic pH (A) Confocal microscopy images of LDs induced by acidic pH (6.5) in breast cancer cell lines MDA-MB-231, MCF7, and T47D. Perilipin 2 (PLIN2, red), Nile Red (green), and DAPI (blue). (B) High-magnification image of LDs induced by acidic pH in T47D cells. (C) Quantification of LDs (green puncta) from MCF7, MDA-MB-231, and T47D. ****p = 0.0001, **p = 0.025, and *p = 0.029. Representative results from three independent experiments and three or more replicates per experiment. (D) Transmission-electron-microscopy image from MCF7 cells grown in pH 6.5 media (N, nucleus; M, mitochondria; ER, endoplasmic reticulum). (E–G) Triglyceride levels from MCF7, T47D, and MDA-MB-231 cells grown at pH 7.4 versus 6.5 media. Unpaired t test, p < 0.0001. (H) Western blot analysis showing upregulation of PLIN2 in low-pH treated cells. (I) Western blot showing the purity of LD-enriched fractions isolated from acid-treated MCF7.

After as few as 24 h of growth in low pH (<pH 6.8) media, breast cancer (and other) cells accumulate LDs, or adiposomes. As shown in Figures 1A and 1B, these droplets are intensely stained with Nile Red, a fluorescent dye that accumulates in neutral lipids. In addition, these LDs stain for the LD coat protein perilipin 2 (PLIN2) as a marker for LDs (Figure 1A). Although PLIN2 is widely disbursed in the cytoplasm at neutral pH, it co-localizes with Nile-Red-positive punctae in MCF7, T47D, and MDA-MB-231 cells under acidic conditions. Quantification of LDs showed a significant number of Nile-Red-positive droplets present in low-pH cultured cells as well as an increase in the number of larger-sized droplets (Figures 1C and S1A–S1C). The average area of LDs was significantly higher in cells cultured at pH 6.5 compared with cells at pH 7.4. Transmission-electron-microscopy imaging validated the presence of LDs in acid-treated cells (Figure 1D). We confirmed that Nile Red staining is specific to LDs and that it does not stain any other cellular organelles such as lysosomes by co-staining cells with lysosome markers (LAMP2 antibody and lysotracker dye) and Nile Red (Figure S2). To further validate whether the acid-induced LD accumulation results in increased lipid content of cells at acidic pH, we compared TG levels of cells grown at pH 7.4 and 6.5. We identified that total TGs, the major lipid species in LDs, are significantly elevated in cells at pH 6.5 compared with 7.4 (Figures 1E–1G). Additionally, there was an induction of PLIN2 protein in cells grown under acidic pH (Figure 1H). We observed that the accumulation of LDs was first visible within 6–8 h of exposure to acid pH and increased with duration of treatment across a large number of cell lines, including breast (MCF7, MDA-MB-231, T47D, MCF10-DCIS), lung (A549, H460), and melanoma (A375) (Figure S3), suggesting that this is a universal stress response to extracellular acidosis. Cytoplasmic lipid accumulation is pH dependent, as only cells cultured at an extracellular acidification (pHe) of 6.8 or lower had a significant increase in LD number. The process was also reversible, as LDs subsequently disappeared beginning 12 h following re-introduction of cells to pH 7.4, implying that these LDs are highly dynamic organelles.

To better characterize the composition of these droplets, we isolated LD-enriched fractions from cells grown in media at pH 6.5 for 72 h using density-gradient ultracentrifugation (Ding et al., 2013). The top layer of the gradient that formed after ultracentrifugation was LD enriched (Figure S1D; STAR Methods). Cytosolic and membrane fractions were also collected at different stages of the isolation protocol to determine the purification efficiency. As shown in Figure 1I, PLIN2 was enriched in LD fractions without appreciable contamination from cytosolic (GAPDH) or membrane (Na^+^/K^+^ ATPase) fractions, as demonstrated by western blots for marker proteins.

### LD formation is associated with increased FA synthesis

**Figure 2.**
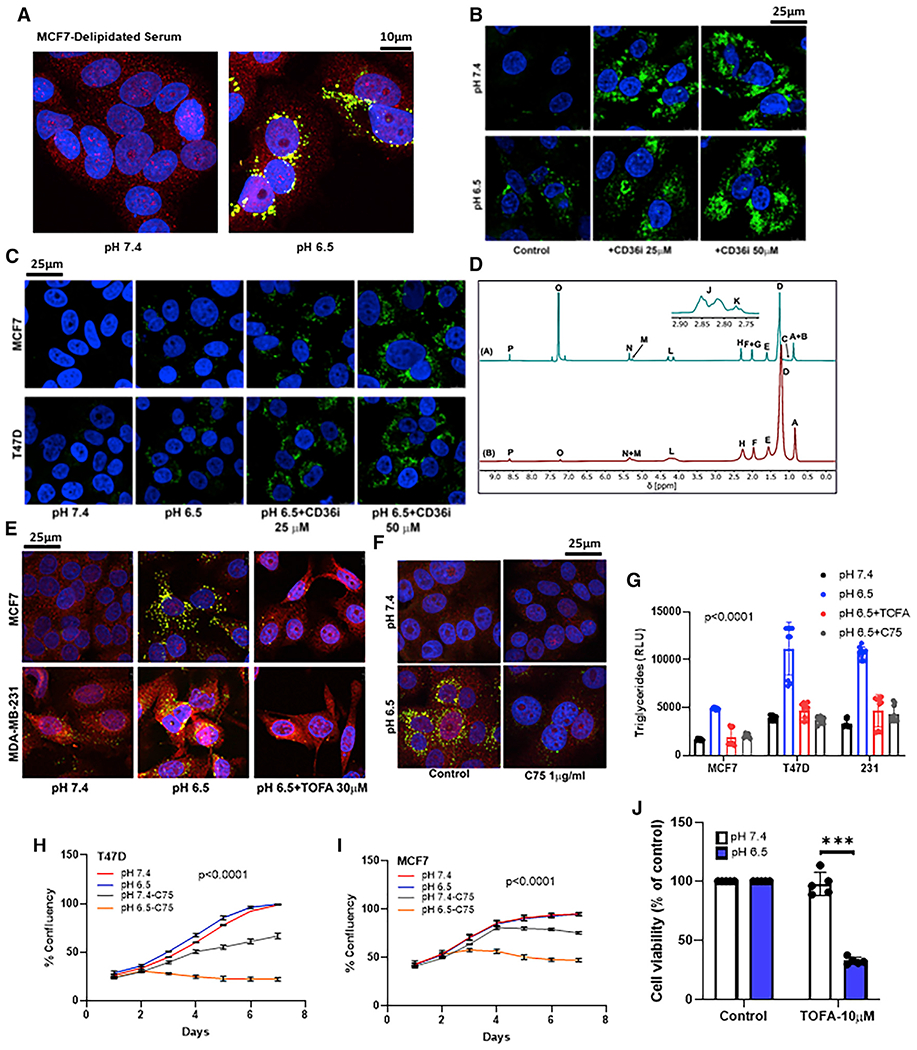
Acid-induced LD accumulation is associated with increased FA synthesis (A) LDs accumulate in cells when grown in delipidated serum under low pH. Confocal microscopy images of LDs induced by acidic pH (6.5) in MCF7 cells. PLIN2 (red), Nile Red (green), and DAPI (blue). (B and C) CD36 inhibition using SSO (25 and 50 μM) did not abrogate LD accumulation in MDA-MB231, MCF7, and T47D cells. (D) ^1^H [A] and ^2^H [B] NMR spectra of the LDs from T47D cells. (A–P) Labeling on each spectrum represents (A) non ω-3 methyl; (B) partial ω-6 methyl; (C) ω-3 methyl; (D) aliphatic chain (methylene); (E) α3 aliphatic (-CH_2_-CH_2_-COO^−^); (F) monounsaturated allylic; (G) polyunsaturated allylic; (H) all α2 aliphatic (-CH_2_-CH_2_-COO^−^); (I) DHA α2 and α3; (J) other bis-allylic; (K) linoleic acid bis-allylic; (L) *sn-1*, *sn-3* of esterified glycerol; (M) *sn-2* of esterified glycerol; (N) olefinic; (O) chloroform; and (P) pyrazine standard. (E and F) FA synthesis inhibition by TOFA (E) and C75 (F) significantly reduced LD formation at low pH. PLIN2 (red) and Nile Red (green). (G) TG levels were significantly lower in C75- and TOFA-treated cells at low pH compared with cells treated with acidic media alone. p < 0.0001, one-way ANOVA. (H and I) C75 (10 μg/mL) inhibited cell growth at low pH (6.5). Cell growth assessed by image-based live-cell analysis system (Incucyte). Two-way ANOVA, p < 0.0001. (J) TOFA (10 μM) inhibited cell viability at low pH (MTT assay). t test, ***p = 0.00016, n = 5.

First, we determined whether cells take up lipids from culture media or whether the lipids are generated *de novo*. Surprisingly, cells accumulated large amounts of lipids when cultured in delipidated serum containing media (Figure 2A), suggesting that these lipids are derived from endogenous *de novo* sources rather than enhanced lipid transport from exogenous sources/media. To further confirm this observation, we inhibited the lipid transporter CD36 (FA translocase) using the irreversible inhibitor sulfosuccinimidyl oleate (SSO). Interestingly, CD36 inhibition did not reduce the accumulation of LDs in acidic pH. On the contrary, there was an increase in LD accumulation in the presence of a CD36 inhibitor in a dose-dependent manner, irrespective of the pH treatment, confirming that the source of lipids that accumulate under acidic growth conditions is not exogenous (Figures 2B and 2C). These data are in contrast to a recent study describing the source of lipids as exogenous, driven by TGFβ2 autocrine signaling to activate FA uptake through translocation of CD36 in several cancer cell lines, notably not breast, suggesting context-dependent mechanisms of lipid metabolism (Corbet et al., 2020).

Next, we determined whether evidence of *de novo* lipogenesis can be detected by positional isotopomer analysis using ^2^H NMR. For this, we cultured T47D cells in 25% deuterium-containing media at pH 6.5 for 72 h to induce accumulation of LDs and isolated LDs using density-gradient centrifugation following standard protocols (Ding et al., 2013). As shown in Figures 2D, ^2^H NMR spectra demonstrated significant incorporation of ^2^H label into lipid. LDs derived from T47D were mainly composed of TGs, with 20% of the total FAs produced via *de novo* lipogenesis. *De novo* lipogenesis is encoded directly by methyl group enrichment; therefore, ^2^H enrichment as estimated by MR is a robust metric of lipogenic contributions to the total pool of fats (Duarte et al., 2014). Taken together, the NMR data presented here provide strong evidence for *de novo* lipogenesis under acidic growth conditions, similar to the acidic microenvironment of solid tumors.

Further, we assessed whether the accumulated LDs are physiologically significant or whether this process is relevant to cell survival under acid stress. To address this initially, we explored whether *de novo* FA synthesis contributes to LD formation under low pH. To investigate this, we incubated cells (MCF7 and MDA-MB-231) at either pH 7.4 or 6.5 and inhibited AcCoA carboxylase (ACC1) with TOFA or FA synthase (FAS) with C75. ATP-dependent carboxylation of AcCoA to form malonyl-CoA by ACC1 is the first committed step in *de novo* FA synthesis. This is also the rate-limiting step for both synthesis and elongation of FAs. Indeed, ACC1 inhibition reduced accumulation of LDs under low pH. As shown in Figure 2E, inhibition of ACC1 by TOFA (10 μM) inhibited acid-induced LD accumulation in both MCF7 and MDA-MB-231 cells. These data suggest that low pH induces *de novo* FA synthesis and that these newly synthesized lipids are stored in LDs. In prior studies, the FAS inhibitor C75 was observed to inhibit acid-induced autophagy (Wojtkowiak et al., 2012), and, in the current study, C75 also strongly inhibited acid-induced LD formation (Figure 2F). Similarly, TG levels were significantly lower in C75- and TOFA-treated cells (Figure 2G). Indeed, cell growth was inhibited by C75 and, in a pH-dependent fashion, in both MCF7 and T47D cells, revealing an acid-selective therapeutic vulnerability (Figures 2H and 2J). Collectively, these results suggest that accumulation of LDs is associated with increased FA synthesis and that this is an adaptive mechanism critical for cell survival under acidic growth conditions.

The rapid, robust, and large increase in LDs across a variety of cell lines, and the observation that PLIN2 levels are maintained even under normal pH (non-stressed) conditions (Figure 1A) suggest that this process must be physiologically significant. Three major questions remain. (1) How is the extracellular-acidosis signal transduced into the cytoplasm? (2) What is the source of the large lipid pool? (3) Is this process relevant to cell survival under acid stress? Below, we have addressed these questions sequentially.

### OGR1 is the major acid-signal-transducing mechanism

LD accumulation occurs rapidly with acid exposure (as early as 6–8 h) and can be reversed under neutral pH, suggesting that this event is dependent on the continuous presence of signal. We thus asked whether LD generation was triggered by pHe simply leading to a decrease in the intracellular pH (pHi). Previous studies in these cells have shown that pHi decreases by only ca. 0.2 pH units when the pHe was decreased from 7.4 to 6.8 (Persi et al., 2018). To investigate this further, we substituted medium NaCl with NaGluconate, which reduces the Cl^−^ gradient and leads to higher steady-state pHi at a given pHe. Under these conditions, reducing pHe in NaGluconate buffer also resulted in accumulation of LDs (Figure S4A), even though the pHi was higher. This is consistent with previous observations suggesting that LD formation was not induced by cytoplasmic acidification (Barba et al., 1999). Hence, we then investigated the role of plasma-membrane acid sensors.

**Figure 3.**
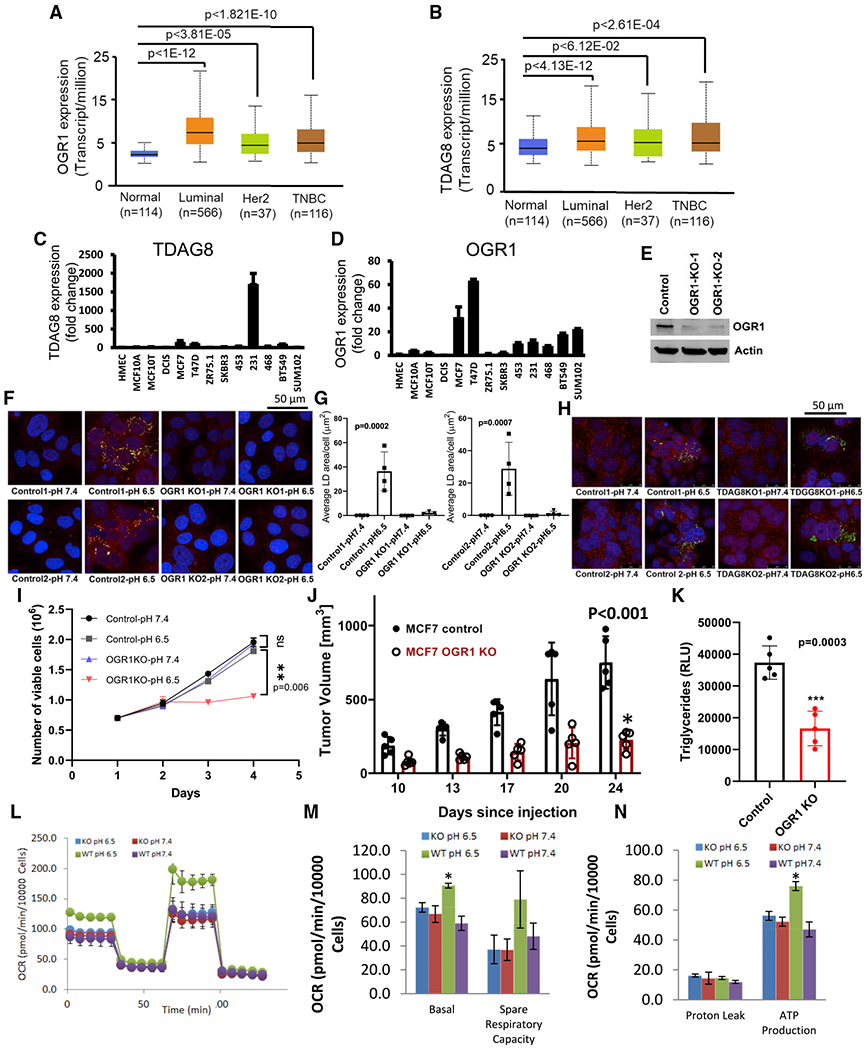
OGR1 might be a major upstream transducer of acid signaling leading to LD formation (A and B) Relative expression of OGR1 and TDAG8 in normal breast tissue and different subtypes of TCGA breast carcinoma samples. (C and D) Expression patterns of OGR1 and TDAG8 in breast cancer cell lines (mRNA fold change). (E) Expression of OGR1 in MVCF7 OGR1-KO cells as seen by western blots. (F–H) OGR1 KO cells show ablation of LD accumulation under low pH (F and G) while it is not affected by (H) TDAG8 depletion. (G) Quantification of LDs, One-way ANOVA. PLIN2 (red), Nile Red (green), and DAPI (blue) in (F) and (H). (I–N) OGR1 depletion affects cell growth and mitochondrial respiration at low pH. (I) Growth of OGR1-KO cells is significantly affected at low pH (6.5) compared with control cells at pH 6.5 (p = 0.006, two-way ANOVA). Data shown are viable cell counts using trypan blue at indicated time points. (J) Tumor growth of MCF7 control cells compared with OGR1-KO cells when implanted in the mammary fat pads of NSG mice. p < 0.001, t test for 24-day data point. (K) TG levels are significantly lower in tumors derived from OGR1-KO cells compared with control. Unpaired t test, p = 0.0003. (L) Representative results of a mitochondrial stress test that measures oxygen consumption rate, following sequential additions of oligomycin, FCCP, and rotenone/antimycin A using extracellular flux analyzer (Agilent). MCF7-control cells grown under low pH exhibited elevated oxygen consumption rate (OCR). OGR1-KO cells did not show pH-induced changes in OCR. (M and N) Basal respiration, spare respiratory capacity (M), and ATP production (N) through mitochondrial metabolism is elevated in MCF7-control cells grown under low pH compared with control cells at pH 7.4, while OGR1-KO cells do not show a pH-induced change in metabolism. *p < 0.05. Data shown represents one of three biological-replicate experiments each with 5 technical replicates.

First, we examined the expression of different acid sensors in human breast tumor specimens compared with normal mammary tissue and identified OGR1 (GPR68) and TDAG8 (GPR65) as being highly expressed in different subtypes of invasive breast carcinomas (Figures 3A and 3B). Assessment of these two acid sensors across the four major breast cancer subtypes (PAM50 classifications) revealed that both OGR1 and TDAG8 were significantly higher in cancers compared with normal breast tissue (Figures 3A and 3B), wherein OGR1 appeared to be more strongly associated with breast cancer disease progression compared with TDAG8 (Figures S4B and S4C). Consistent with these findings, analysis of histological subtypes confirmed overexpression of both OGR1 and TDAG8 in human breast tumor specimens compared with normal breast tissue (Figures S4D and S4E). Interestingly, GPR4 was not overexpressed in breast tumors compared with normal breast tissue (Figures S4F and S4G). Additionally, quantitative PCR (qPCR) analyses showed that both TDAG8 and OGR1 are highly expressed in a panel of breast cancer cell lines compared with non-malignant breast epithelial cells. As shown in Figures 2C and 2D, highly invasive and acid-tolerant triple-negative breast cancer (TNBC) MDA-MB-231 cells express significantly higher levels (>750-fold) of TDAG8, while less invasive and estrogen receptor-positive MCF7 and T47D cells have very high levels of OGR1. To test the functional significance of these receptors, we decided to deplete their levels using small interfering RNA (siRNA) approaches. However, both OGR1 and TDAG8 were potently induced by low pH, and hence, siRNAs were not effective in depleting their expression (Figures S5A and S5B). Consequently, we generated cell lines where major acid receptors TDAG8 and OGR1 were depleted using CRISPR-Cas9 technology following standard/published protocols (STAR Methods) (Sanjana et al., 2014). We confirmed positive clones by DNA sequencing, which showed a complete knockout of TDAG8 and OGR1 (Figure S5C), as well as western blots, which showed significant reductions of OGR1 protein (Figures 2E and S5D). We then assessed the formation of LDs under low pH in TDAG8 and OGR1 knockout MCF7 cells compared with control cells by confocal microscopy after staining with Nile Red and PLIN2. As shown in Figures 2F and 2G, we observed significantly lower levels of LDs in OGR1 knockout cells (OGR1-KO) compared with control cells at low pH. In contrast, TDAG8 depleted MCF7 cells did not show any reduction in LD accumulation (Figure 2H). Likewise, we knocked out OGR1 in T47D cells, which also resulted in abrogation of LD formation under acidic conditions (Figures S5E and S5F). In addition, stable cell lines overexpressing OGR1 generated by transfecting pCMV-OGR1 in MCF7 cells also exhibited accumulation of LDs upon acidosis (Figures S5G–S5I). These combined data suggest that OGR1 is the major receptor that is involved in transducing the acid signal leading to acid-induced accumulation of LDs in MCF7 cells.

### Depletion of OGR1 affects cell growth under acidosis

**Figure 4.**
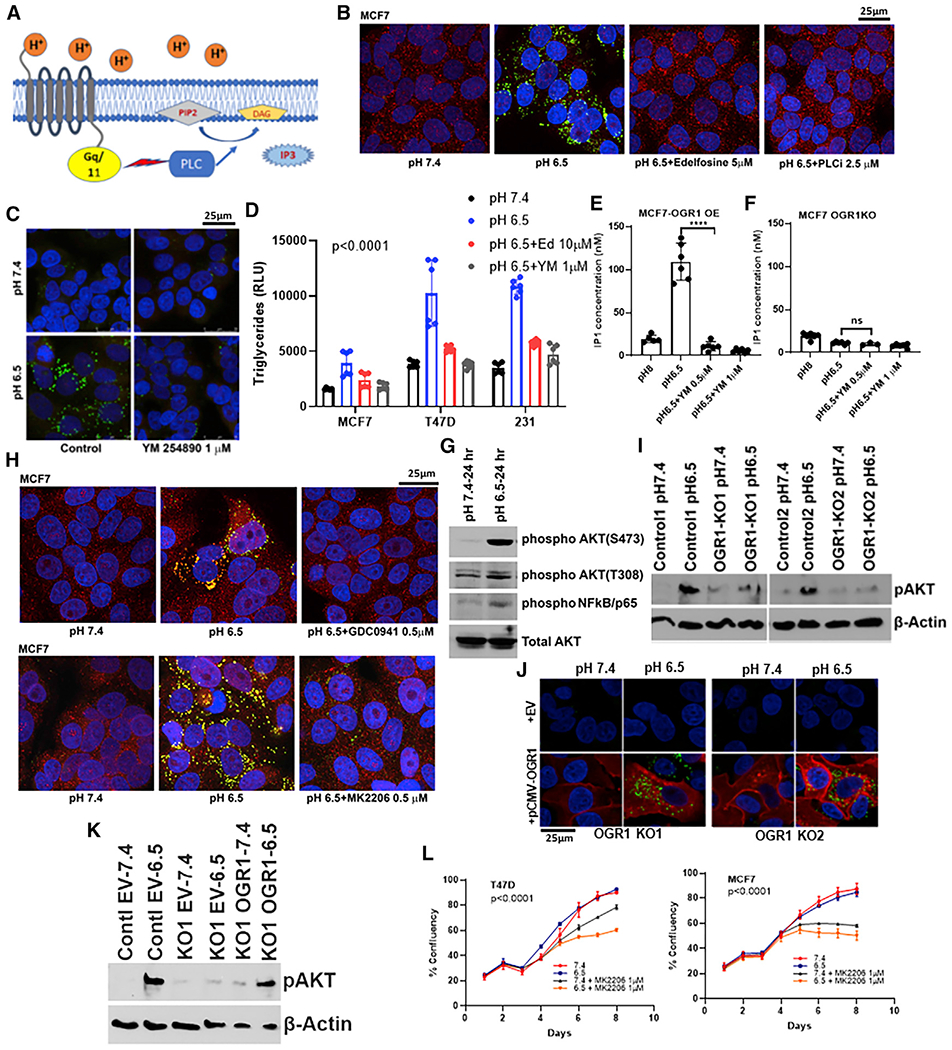
Lipid-droplet formation under low pH is affected by inhibition of downstream signaling of PL-C and PI3Kinase/Akt pathway (A) Schematic showing signaling through OGR1 receptor. (B) LD accumulation is inhibited by pharmacological inhibitors of PL-C, edelfosine (5 μM), or U73122 (2.5 μM) under low pH in MCF7 cells. (C) MCF7 cells treated with YM254890, an inhibitor of G_q/11_-coupled GPCR signaling, affected LD accumulation. PLIN2 (red), Nile Red (green), and DAPI (blue). (D) Triglyceride levels from cells grown in pH 7.4, 6.5, or 6.5 media along with inhibitors of OGR1 downstream signal mediators PL-C and G_q/11_. Acidic-pH-induced increases in TG levels were abrogated by inhibitor treatments. p < 0.0001, one-way ANOVA analysis for each cell line. (E) Acidic pH induced OGR1-mediated Gq/11 activation as seen by IP1 assay. IP1 levels in OGR1-overexpressing MCF7 cells were higher at pH 6.5 compared with 8 and were inhibited by G_q/11_ inhibitor (T test, p < 0.0001). (F) OGR1-depleted cells did not induce IP1 upon low-pH treatment, and this was not significantly altered by G_q/11_ inhibitor. t test, not significant. (G) Acidic pH induces Akt phosphorylation (residues S473 and T308) and phosphorylation of NFkB, the downstream target of Akt activation, as seen by western blot analyses. (H) Inhibition of PI3K using GDC0941 (0.5 μM) or Akt inhibition by MK2206 (0.5 μM) resulted in significantly lower levels of LDs under low pH in MCF7 cells. (I) Acid-induced Akt phosphorylation (S473) was abrogated in OGR1-depleted cells, as seen by western blot analysis. (J) Ectopic expression of OGR1 rescued acid-induced LD accumulation in OGR1-depleted cells. OGR1-KO cells were transfected with either empty vector control or Myc-tagged OGR1 and subsequently induced with pH 7.4 or 6.5 media (Nile Red, green; Myc, red; DAPI, blue). (K) Akt phosphorylation (S473) was rescued in OGR1-depleted cells by ectopic expression of OGR1. (L) Akt inhibition is selectively cytotoxic at acidic pH. Akt inhibitor MK0026 treatment decreased growth rate in low-pH culture conditions compared with neutral pH. p < 0.0001, two-way ANOVA.

To investigate the effect of OGR1 depletion on cell growth, we assessed the growth of control as well as OGR1-KO MCF7 cells grown in acidic culture media. As shown in Figure 4I, the growth rates of OGR1-KO cells were significantly lower than the control cells under acidic growth conditions. There was no significant difference in growth rates at pH 7.4. To test this *in vivo*, we implanted these cells in the mammary fat pads (MFPs) of NSG mice (n = 10) and assessed tumor growth by caliper measurements. As shown in Figure 4J, tumor volume was significantly smaller in tumors derived from OGR1-depleted MCF7 cells compared with tumors derived from control MCF7 cells. In addition, levels of TGs, a major component of LDs, were significantly lower in OGR1-depleted tumors compared with controls (Figure 4K).

In addition, the oxygen consumption rate (OCR) of MCF7-control cells grown in acidic media was elevated compared with cells grown in normal pH media, consistent with previous studies (Persi et al., 2018). However, OGR1-depleted cells did not show this increase in OCR, suggesting that the OGR1 signal induces respiration and perhaps that lipids stored in LDs could be a fuel source in MCF7-control cells under acidosis (Figure 4L). As shown in Figures 4M and 4N, basal respiration, ATP production (p < 0.05), and spare respiratory capacity were higher in control cells grown under acidic conditions compared with control cells at pH 7.4 or OGR1-KO cells at either pH.

### OGR1 downstream signaling is involved in acid-induced LD formation

To further investigate whether activation of OGR1 was sufficient for the induction of LD formation, we employed Ogerin, a known small-molecule agonist of OGR1 (Huang et al., 2015). OGR1 is completely inactive at alkaline pH; however, Ogerin can activate OGR1 at this pH. As shown in Figures S6A and S6B, a significant accumulation of LDs at alkaline pH was observed in the presence of Ogerin (5 and 10 μM), suggesting that OGR1 activation alone was sufficient to trigger LD accumulation in MCF7 and T47D cells. Next, we explored acid-induced signaling downstream of OGR1 activation leading to LD accumulation. OGR1 primarily couples to G_q/11_; as such, ligand (H^+^) binding to OGR1 triggers activation of phospholipase C (PL-C), which stimulates formation of the second messenger, inositol 3,4,5 trisphosphate (IP3) (Figure 4A). We assessed the role of PL-C in transducing the signal from OGR1 by treating cells with inhibitors of PL-C, edelfosine or U73122 (Leitner et al., 2016), at low or neutral pH and staining with Nile Red. As shown in Figures 4B and S6C, both edelfosine (5 μM) and PL-Ci/U73122 (2.5 μM) inhibited accumulation of LDs at low pH in MCF7 and T47D cells. Additionally, we treated cells with 1 μM YM 254890, a cyclic peptide of bacterial origin that is a direct, potent inhibitor of G_q/11_ signaling. As shown in Figures 4C and S6D, LD accumulation was inhibited by YM 254890 in both MCF7 and T47D cells. Similarly, TG levels from edelfosine- or YM 254890-treated cells at pH 6.5 was significantly lower compared with TG levels from cells that were grown in acidic pH (6.5) with LD accumulation (Figure 4D). To confirm that the inhibition of G_q/11_ by YM 254890 results in decreased second messenger (IP3) formation, we measured IP1, a surrogate of IP3, using the IP-One Gq HTRF assay (Cisbio). As shown in Figure 4E, there was an increase in IP1 levels upon low pH treatment in cells ectopically expressing OGR1, and this pH-induced IP1 induction was abrogated by G_q/11_ inhibition. pH-dependent IP1 production was not observed in OGR1-depleted cells, and IP1 levels were not significantly altered by the Gq inhibitor (Figure 4F), indicating a role for OGR1 in inducing G_q/11_ signaling. Taken together, these data confirm the role of G_q/11_ axis in LD accumulation downstream of OGR1 activation by acidic pH.

Extracellular signals transduced by G_q/11_-coupled GPCRs are known to activate PI3 kinases (PI3Ks) leading to Akt phosphorylation. Indeed, MCF7 cells incubated at acid pH showed robust increased phosphorylation of Akt at S473 and a weaker induction at T308, as well as phosphorylation of the p65 subunit of nuclear factor κB (NF-κB), consistent with activation of the Akt pathway (Figure 4G). We then investigated whether inhibiting PI3K signaling pathways would inhibit the formation of LDs under low pH. MCF7 and T47D cells were treated with PI3Kα inhibitor GDC-0941 (0.5 μM) or the Akt inhibitor MK2206 (0.5 μM). As shown in Figure 4H, both GDC-0941 and MK2206 treatment resulted in inhibition of LD formation under low pH, indicating that the activation of the PI3K-Akt signaling axis downstream of pH-sensing GPCRs is critical for LD generation. OGR1-depleted cells failed to activate Akt phosphorylation upon acid treatment, confirming the role of OGR1 in transducing this signaling axis (Figure 4I). In addition, we could rescue Akt activation and lipid phenotype under acidosis by ectopic expression of OGR1 in OGR1-KO cells (Figures 4J and 4K). Moreover, Akt inhibition was selectively cytotoxic at low pH conditions, suggesting a key role of this signaling axis in cell survival (Figure 4L).

### Acid-induced LD accumulation is dependent on autophagy

**Figure 5.**
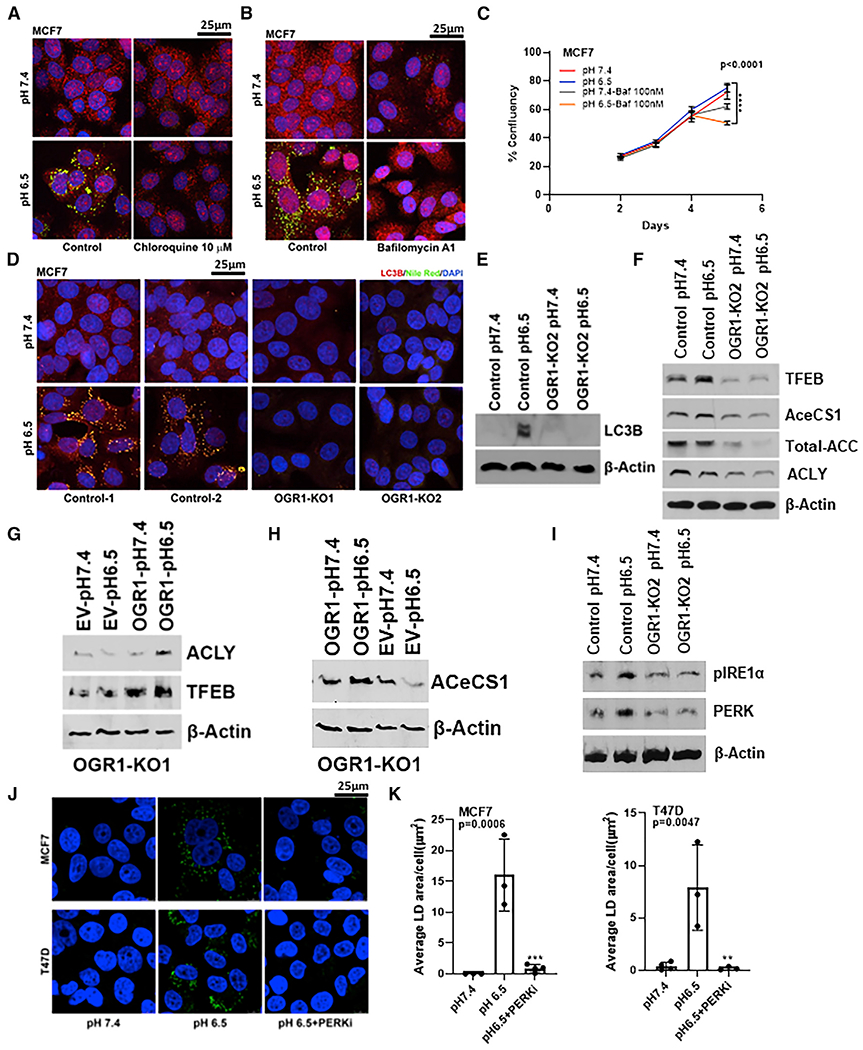
Acid-induced LD accumulation is dependent on autophagy (A and B) LD accumulation under low-pH conditions was significantly inhibited when MCF7 cells were treated with autophagy inhibitors (10 μM chloroquine or 100 nM bafilomycin A1). PLIN2 (red), Nile Red (green), and DAPI (blue). (C) Bafilomycin treatment resulted in significant inhibition of cell growth under acidic-growth conditions. Cell growth assessed by image-based live-cell analysis system (Incucyte). p < 0.0001, one-way ANOVA for last data point. (D) Acid-induced autophagy is inhibited in OGR1-KO MCF7 cells. LC3B (red), Nile Red (green), and DAPI (blue). (E and F) OGR1-KO cells failed to induce LC3 at low pH (E) and OGR1-KO cells express lower levels of TFEB, ACC, ACeCS1 (ACSS2), and ACLY (F) compared with control cells under pH 6.5, as demonstrated by western blot analysis. (G and H) ACLY and ACeCS1 induction and restoration of TFEB indicative of lipogenic phenotype in OGR1-depleted cells expressing OGR1. (I) OGR1-depleted cells failed to upregulate ER stress sensors. (J and K) Treatment with PERK inhibitor AMG44 abrogated LD accumulation under acidic-growth conditions. Nile Red (green). (K) Quantification of LD using Definiens. MCF7 p < 0.0001; T47D p = 0.0211 (one-way ANOVA).

Previous studies from our lab showed that one of the major survival mechanisms adopted by tumor cells under low pH is chronic activation of autophagy (Wojtkowiak et al., 2012; Wojtkowiak and Gillies, 2012). Hence, we next explored whether acid-induced LD accumulation is dependent on the ability of cells to undergo autophagy. For this, we treated MCF7 cells with autophagy inhibitors bafilomycin A or chloroquine. Bafilomycin A1, a potent inhibitor of the vacuolar H^+^ ATPase, inhibits fusion between autophagosomes and lysosomes and prevents maturation of autophagic vacuoles. Chloroquine is a lysosomotropic weak base that raises lysosomal pH, inhibiting autophagic degradation in lysosomes. Acid-induced LD accumulation was profoundly inhibited in the presence of chloroquine (10 μM) or bafilomycin A1 (100 nM), suggesting that LD genesis is coupled to autophagy (Figures 5A and 5B). Consistent with this, we observed that inhibition of autophagy was selectively cytotoxic in cells grown under low pH, suggesting that autophagy and lipogenesis are survival mechanisms under acidosis (Figure 5C). As OGR1-KO cells did not accumulate LDs when exposed to low pH, we also assessed whether OGR1 cells underwent acid-induced autophagy. Acid-treated control cells showed the presence of the autophagy-related marker LC3B-positive punctae by immunofluorescent staining (Figure 5D), as well as by western blot analysis (Figure 5E), while OGR1-KO cells had lower LC3B or absent punctae at acidic pH. In addition, MCF7 control cells treated with pH 6.5 media showed an increase in transcription factor EB (TFEB), the master regulator of autophagy and lysosomal biogenesis, while OGR1-depleted cells expressed lower levels of TFEB at pH 6.5 (Figure 5F). There was also a concomitant decrease in the levels of lipogenic enzymes ACSS2, ACC, and ACLY, as seen by western blots in OGR1-depleted cells (Figure 5F). Ectopic expression of OGR1 in OGR1-depleted cells rescued low-pH-induced expression of ACeCS1, ACLY, and TFEB (Figures 5G and 5H). Further, control cells, when exposed to low pH growth conditions, induced expression of endoplasmic reticulum (ER) stress sensors such as phosphor-inositol-requiring enzyme-1α (IRE1α) and protein kinase R-like ER kinase (PERK), indicating an ER stress response (Figure 5I). OGR1-depleted cells failed to upregulate ER stress sensors, suggesting that pH sensing by OGR1 may also be required for triggering an ER stress response. Similarly, when treated with a PERK inhibitor (AMG44, 1 μM), LD accumulation was inhibited in MCF7 and T47D cells (Figures 5J and 5K). It is tantalizingly possible that ER stress is the priming signal that triggers autophagy under acidosis. ER stress has been shown to induce nuclear translocation of TFEB as part of the integral stress response where PERK regulates nuclear translocation of TFEB (Martina et al., 2016). In addition, PERK is shown to regulate lipid metabolism and phospholipid biosynthesis (Bobrovnikova-Marjon et al., 2008). Taken together, these results suggested that acid-induced ER stress and autophagy are triggered by acid-sensing GPCR OGR1 and that these processes are required for acid-induced LD formation.

### Ketogenic amino acids are the source of lipids stored in LDs

**Figure 6.**
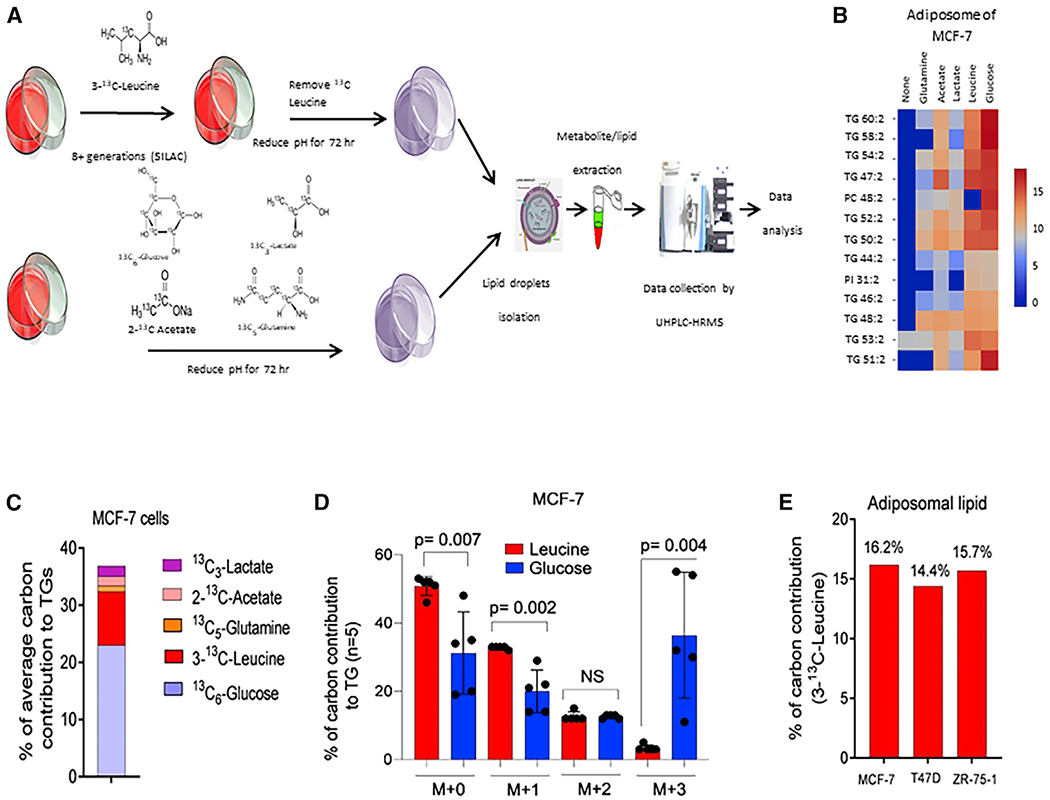
Leucine and glucose metabolism contribute to lipid stores in LDs (A) A typical workflow of ^13^C-labeled isotope distribution in LDs of breast cancer cells. This workflow includes treatment of breast cancer cells with ^13^C-labeled glucose, lactate, glutamine, acetate, and leucine, LD isolation, non-targeted lipidome extraction, and UHPLC-HRMS-based mass spectrometry data analysis and processing. (B) Heatmap showing relative abundance of ^13^C in different species of TGs and phospholipids from LDs of cells labeled with glucose, lactate, glutamine, acetate, and leucine. (C) Comparative labeling activity of ^13^C-labeled glucose, lactate, glutamine, acetate, and leucine with TGs. (D) Comparative carbon contribution between glycerol backbone (M+3) and acyl chains (M+1 and M+2) by leucine and glucose in MCF-7 cells. The p values were calculated based on unpaired t test. 5 independent TG molecules were used for statistical analysis. (E) Total labeling estimation for ^13^C-labeled leucine in lipids stored in LDs of different breast cancer cell lines.

To investigate the source of the lipid precursors and to identify the lipids stored in LDs, we next performed ultra-high-pressure liquid chromatography coupled to high-resolution mass spectrometry (UHPLC-HRMS) analyses of purified LDs. To identify the major carbon source of the lipid precursors in LDs, we employed ^13^C tracer analysis using a variety of carbon sources, [UL] ^13^C_6_ glucose, 2-^13^C acetate, [UL]^13^C lactate, and [UL]^13^C glutamine, as direct metabolic substrates that were added to the media upon acidification and incubation for 72 h (Figure 6A). Since cells can consume and trap acetate as AcCoA via AcCoA synthetase-2 ACSS2, especially under hypoxia and serum deprivation (Schug et al., 2015), we investigated the possibility of acetate as a carbon source. Glutamine is a well-known carbon and nitrogen source for anabolic processes (Hosios et al., 2016). Glucose can contribute to lipid synthesis by either forming the glycerol backbone (glycerol-3 phosphate) or the FA precursor via AcCoA. Finally, lactate can be converted to pyruvate and hence can also populate the AcCoA pool. For these experiments, ^13^C label incorporation was determined by UHPLC-HRMS from LDs isolated after labeling cells for 72 h in the presence of labeled carbon source in media specifically depleted of that carbon source and containing delipidated serum at pH 6.5.

As an alternative to the direct uptake of precursors from the media, we also investigated endogenous sources, such as autophagic breakdown products. Proteins were labeled by stable isotope labeling by amino acids in cell culture (SILAC). Leucine-free media were substituted with 0.8 mM 3-^13^C leucine, and cells were grown in this culture media for 8 generations. At the time of experiment, the media were replaced with complete label-free media during the 72 h incubation at acid pH. The rationale for leucine labeling was to test whether breakdown of ketogenic amino acids (e.g., during autophagy) may be stored as lipids, which may preserve this important energy source to meet future biosynthetic and energy demands and to avoid toxicity.

As shown in Figures 6B and 6C, the major contributors to ^13^C labels in isolated LDs were glucose and leucine. ^13^C label incorporation from [UL] ^13^C glucose was primarily incorporated into the glycerol backbone, with lesser contributions into lipid acyl chains (Figure 6D), whereas the majority of the label from leucine was incorporated into acyl chains. Acyl carbons (14%–16%) were enriched from 3-^13^C leucine across cell lines incubated at pH 6.5 (Figure 6E). This is considered highly significant, as (1) leucine contained only a single ^13^C label (compared with, e.g., UL-glucose), (2) it is one of two strictly ketogenic amino acids along with lysine; further, and (3) there are 5 additional amino acids whose breakdown results in carbons supplying the AcCoA pool and hence acyl chains: phenylalanine, isoleucine, threonine, tryptophan, and tyrosine. The majority of leucine is metabolized to α-ketoisocaproate (α-KIC) by branched-chain amino-acid (BCAA) aminotransferase enzyme. α-KIC eventually metabolizes to HMGCoA, which is directly cleaved to form AcCoA as an FA precursor (Figure S7). If 3-^13^C leucine was the major carbon source for lipids stored in LDs, half of the acyl-chain carbons coming from leucine will thus be labeled. After labeling with 3-^13^C leucine to steady state and induction with acidic pH, we detected 16.2% of lipid carbons were ^13^C labeled in LDs from MCF7 cells (Figures 6C and 6D) and 14.4% and 15.7% lipid labeled in LDs from T47D and ZR75.1 (Figure 6E) cells. Taken together, our data strongly indicate that ketogenic amino acids arising from the autophagic breakdown of proteins are the major source of carbons in lipids stored in LDs.

### Lipogenic phenotype is associated with poor patient survival

**Figure 7.**
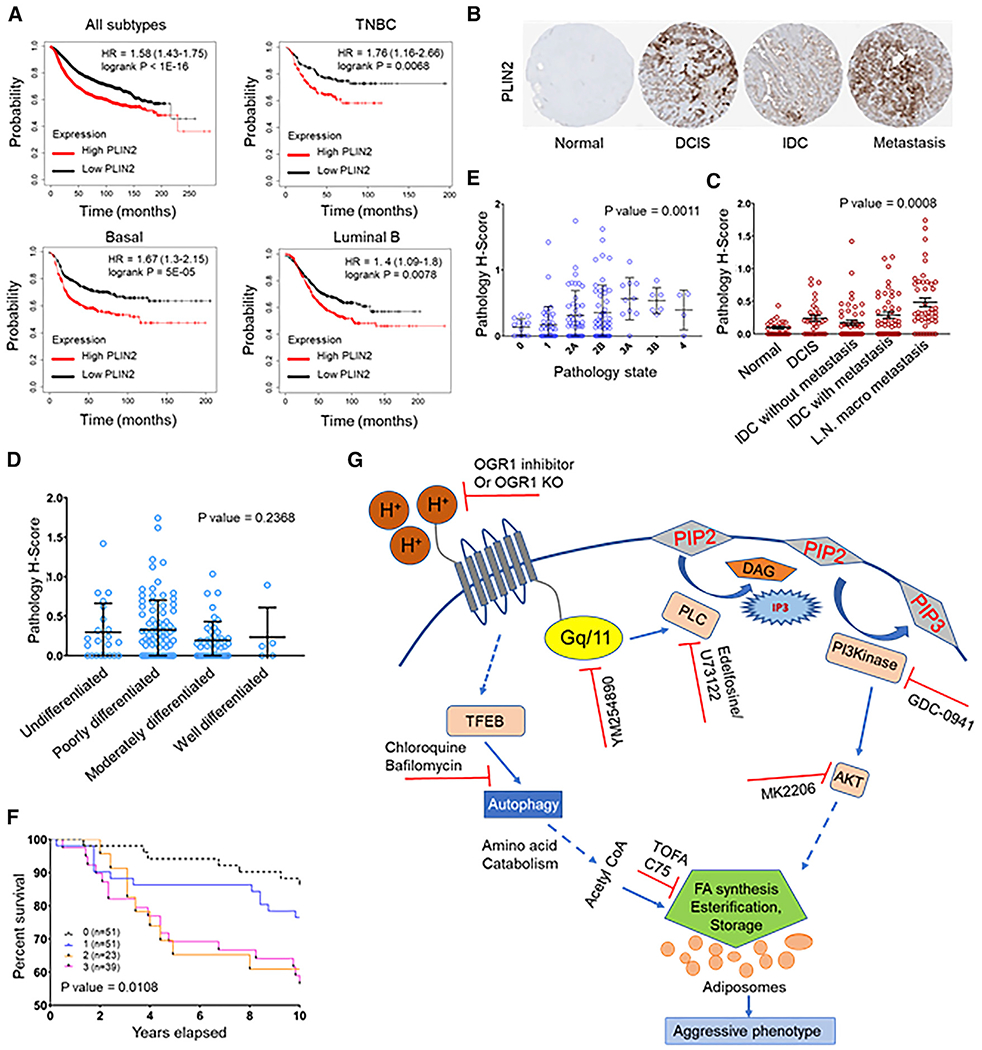
PLIN2 expression is associated with poor patient survival (A) Kaplan-Meier survival plot showing significantly low survival of patients with high PLIN2 expression. High expression of PLIN2 is associated with poor prognosis of patients with TNBC, basal, and luminal B subtype of breast tumors (KM plotter). (B) Representative images from breast cancer progression tissue microarray (TMA) stained for PLIN2. (C) Increase in PLIN2 expression correlates with aggressive/metastatic tumors (p = 0.0008). (D) Expression of PLIN2 did not significantly differ between undifferentiated, poorly differentiated, and well-differentiated tumors (p = 0.2368). (E) PLIN2 levels increases with breast cancer progression, as seen from cores representing different stages (p = 0.0011). (F) KM plot showing significantly low survival of patients with high PLIN2 expression as quantified from the TMA. p = 0.0108. (G) Schematics of the mechanism by which acid-induced LD accumulation occurs in breast cancer cells. Acidosis triggers signaling through acid receptors such as OGR1 and induces autophagy supporting *de novo* lipogenesis and LD accumulation. Acid-induced signal-transduction cascade involves activation of G_q/11_, phospholipase C, PI3K, and Akt. Inhibition of these signaling events using pharmacological inhibitors (red symbols) dampened acid-induced LD formation. Acid induction of LDs occurs even in de-lipidated serum, indicating the endogenous origin of lipids. Further, acidosis induced autophagy though the master regulator TFEB. Blocking autophagy and lipogenesis also blocked LD accumulation. Catabolism of ketogenic amino acids produced during autophagic proteolysis contribute significantly to the carbon pool for FA synthesis and storage as LDs. These events appear to be important for survival under acid stress, as the predominant LD coat protein PLIN2 gene expression is associated with poor survival in breast cancer. (Dotted arrows represent events involving multiple steps.)

It is well established that microenvironments of solid tumors are unequivocally acidic and that this acidic microenvironment promotes the evolution of more aggressive and invasive tumors (Gillies and Gatenby, 2007; Pillai et al., 2019). Further, inhibition of FA synthesis was selectively cytotoxic to cells at acidic pH (Figures 2E–2J). Moreover, attenuating signaling from OGR1 also affected cell growth at low pH (Figure 3I). Thus, a lipogenic phenotype might be indicative of a tumor that is under acidic stress and hence be prone to evolve into more aggressive cancer. As shown in Figure 7A, data obtained by survival analysis using a Kaplan-Meier (KM) plotter from breast cancer patients indicate that high expression of PLIN2 is associated with poor patient survival in all histological subtypes, including triple negative, basal, and luminal B (log rank p = 0.002 for TNBC, p = 0.0002 for luminal B, p = 0.00061 for basal) (Gyorffy et al., 2010; Nagy et al., 2018). Further, we used immunohistochemistry to assess the levels of PLIN2 in human breast cancer tissue arrays compared with normal breast tissue. For this, we stained a breast cancer progression microarray containing 199 cores, representing different stages of breast cancer, with anti-PLIN2 antibody (Figure 7B). We measured the positivity of each core for PLIN2 expression in cores representing progression of breast cancer. Enhanced expression of PLIN2 correlated with disease progression, from ductal carcinoma *in situ* (DCIS) to invasive ductal carcinoma and to lymph node macrometastases (p = 0.0008) (Figure 7C) and for advanced stages of breast cancer (p = 0.001) (Figure 7D). However, PLIN2 levels were not significantly altered based on the differentiation stages, from undifferentiated to well-differentiated tumors (p = 0.237) (Figure 7E). Survival curves generated using KM analysis revealed that patients with a higher expression of PLIN2 had significantly lower survival (p = 0.01) (Figure 7F).

## DISCUSSION

Acidosis in the tumor microenvironment is a common feature of solid tumors because of altered metabolism, poor vasculature, and hypoxia. Acidosis imparts selection pressure in the tumor and reprograms cancer cells to adapt to the acidic conditions that confer selective advantage for invasion into the stroma and distant metastasis. Our previous studies identified autophagy as a mechanism of adaptation to chronic exposure to acidosis. Acidosis also results in increased *de novo* lipid synthesis and accumulation of lipids as intracellular LDs. While numerous studies have identified intracellular LDs in cancer cells, it has generally been considered an epiphenomenon of metabolic perturbations. Our data suggest that this is a regulated process that might be essential for cell survival under stressful acidic conditions. As protein degradation is a hallmark of autophagy, this may form a nexus between these two pathways of acid adaptation. Interestingly, a recent study in adipocytes demonstrated that differentiated adipocytes catabolized BCAAs, such that leucine and isoleucine from media and/or protein catabolism accounted for approximately one-third of lipogenic AcCoA while proliferating pre-adipocytes used mainly glucose and glutamine for *de novo* lipogenesis (Green et al., 2016). We speculate that similar metabolic reprogramming could occur in the acidic tumor microenvironment, leading to LD accumulation.

The accumulation of LDs in cancer cells under acidic conditions has also been observed by other groups. A recent study (Corbet et al., 2020) suggested that this phenotype is induced during the epithelial-to-mesenchymal transition, where TGF-β2 stimulation under acidic conditions enhanced FA uptake through CD36, resulting in TG storage in LDs. In contrast to this observation, our studies revealed an increase in autophagy under acidic conditions and subsequent *de novo* lipogenesis as the source of lipids stored in LDs (Wojtkowiak et al., 2012). This phenotype is robust even in the absence of exogenous lipids and rapid, as it requires only 6–8 h for LD accumulation upon acidification. Further, we observed that this is a reversible phenotype once cells are placed in neutral pH, suggesting that LD accumulation is a dynamic process induced by acid signal.

Our study identified a direct signaling axis that links extracellular-acid sensing to LD accumulation. Although our studies indicate OGR1 as the major acid sensor in MCF7 and T47D cells that drive acid-induced LD accumulation, it is possible that acid-sensing ion channels (ASICs) or other GPCRs such as TDAG8 could contribute to acid sensing in other breast cancer cell lines, supporting the notion that lipogenic phenotype is an atavistic response to microenvironmental stress induced by acidosis. Many microenvironmental stressors such as oxidative stress, acidosis, nutrient stress, hypoxia, and many oncogenes, as well as GPCR signaling, are known to cause ER stress (Senft and Ronai, 2015; Poplawski et al., 2019). There is accumulating evidence for the regulation of autophagy through PERK-dependent ER stress response and perturbations in calcium homeostasis (Kania et al., 2015; Ma et al., 2014; Margariti et al., 2013). A recent study showed that exogenous expression of OGR1 in an intestinal epithelial cell model activated ER stress and inhibited late-stage autophagy to promote cell survival (Maeyashiki et al., 2020). However, our data suggests that ER stress induced by acidic conditions promoted autophagy and that acid sensing by OGR1 was indeed required for ER-stress-induced autophagy. Moreover, it is possible that ER stress is the priming signal that initiates autophagy under acidosis. As ER is the major Ca^2+^ reservoir in the cell and disturbances in Ca^2+^ homeostasis can also result in ER stress, it is tempting to speculate that Ca^2+^ signaling might play a role in this. Our studies shed light on how acid-stress sensing by OGR1 and the molecular pathways that are activated to alleviate this stress are important for cell survival and growth. Importantly, we provide evidence for lipid accumulation as an adaptive response to acid stress and OGR1 activation.

Recent studies have established reprogramming of lipid metabolism as an emerging hallmark of many cancers, providing opportunities for therapeutic targeting. Many lipid inhibitors are being investigated as anticancer drugs in clinical trials. We observed that inhibitors of lipogenesis, C75, and TOFA selectively inhibited cell viability under acidic-culture conditions. These are weak acids and are prone to ion trapping (Raghunand et al., 2003). Thus, it is not known whether the enhanced sensitivity at acidic pH is due to the criticality of FA synthesis for survival or whether more drug is sequestered at acidic pH via ion trapping. Ion trapping is unlikely, however, based on their lipophilicity; this nonetheless remains to be explored. Regardless of mechanism, these data show that fatty-acid synthase (FAS) inhibitors are more toxic at acidic pH, such as that encountered in tumors, and this may be reflected in their strong anti-tumor activity (Kuhajda et al., 2000; Zaytseva et al., 2018). Further, we demonstrate that high expression of PLIN2, the LD coat protein, a marker of lipid phenotype, was observed to be strongly associated with poor overall survival in breast cancer patients, suggesting that PLIN2 could be useful as a biomarker for aggressive breast tumor types and to identify those that may have a greater response to lipid-synthesis inhibition. As shown in the schematic (Figure 7G), our attempts to characterize the molecular pathways regulating acidosis-induced lipogenic phenotype indicate that the lipid phenotype is a unique metabolic vulnerability that can be targeted therapeutically.

### Limitations of the study

Although the cytotoxicity or cell-growth inhibition is consistent with the hypothesis that OGR1-mediated autophagy/LD induction appears to be a survival mechanism at low-pH growth conditions, we have not explored the potential off-target effects by some of the pharmacological reagents that could also contribute to cell toxicity. Future studies are required to test if “ion trapping” of the drugs affect their efficacy in an acidic environment. In addition to using inhibitors of lipogenic enzymes or PI3Kinase and Akt inhibitors, siRNA/short hairpin RNA (shRNA)-based knockdown of these genes will be informative in this context. Although we observe acid-induced LD accumulation in a large panel of breast cancer cell lines, we have not determined the major acid sensors in cell lines representing more aggressive TNBC subtypes. Our studies provide a strong foundation for future investigations that will lead to a more detailed characterization of the role of OGR1 in ER stress response, autophagy, and lipogenesis in animal models and human breast tumors.

## STAR★METHODS

### RESOURCE AVAILABILITY

#### Lead contact

Further information and requests for resources and reagents should be directed to and will be fulfilled by the lead contact, Smitha Pillai (Smitha.Pillai@moffitt.org).

#### Materials availability

Plasmids generated in this study are available upon request from the lead contact.

#### Data and code availability

- Microscopy data reported in this paper will be shared by the lead contact upon request.
- Lipidomics data are deposited at Zenodo Data (https://doi.org/10.5281/zenodo.6449175) and are publicly available. Link to this data is listed in the key resources table.
- This paper does not report original code.
- Any additional information required to reanalyze the data reported in this paper is available from the lead contact upon request.

### EXPERIMENTAL MODEL AND SUBJECT DETAILS

#### Cell lines and culture conditions

MCF7, T47D, MDA-MB-231, MDA-MB-468, MDA-MB-157, ZR75.1, SKBR3 and BT549 are obtained from ATCC/PSON. Cells were maintained in DMEM supplemented with 10% FBS. Cells were tested for mycoplasma contamination and authenticated using short tandem repeat DNA typing according to ATCC recommendation. For pH treatments, growth medium was further supplemented with 25 mM each of PIPES and HEPES and the pH adjusted to 7.4 or 6.5.

#### *In vivo* mouse studies

Xenograft studies were performed with 6–8 weeks old female NSG mice (Jackson lab) and all animals were maintained in accordance with IACUC standards of care in pathogen-free rooms, in the Moffitt Cancer Center and Research Institute (Tampa, FL) Vivarium. 10 million MCF7 control and MCF7 OGR1 KO cells were injected into the mammary fat pads (MFP) along with equal volume of Matrigel. Mice were weighed and tumors were measured using electronic caliper twice weekly. Statistical analysis was performed using Student t-test and values were considered significant when the p value was <0.05.

### METHOD DETAILS

#### Isolation of lipid droplet enriched fractions

LD enriched fractions were isolated from cells using density gradient ultracentrifugation described by Ding et al., Nature protocols, 2010. The protocol is based on the fact that lipid droplets float on the top of all aqueous gradients after centrifugation due to their low density associated with their core of neutral lipids. Briefly, MCF7 and T47D cells were treated with acidic media (pH 6.5) for 72 h and cells were collected by trypsinization. 180 million cells were pelleted by centrifugation. Cell pellet was disrupted by incubating in a buffer (buffer A) containing 250 mM sucrose to protect the intracellular organelles. The homogenate was subjected to low-speed centrifugation at 3000 × g for 10 min at 4°C to remove nuclei, cell debris and unbroken cells. The post-nuclear supernatant, PNS, was transferred to ultracentrifuge tubes and a buffer (buffer B) containing 20 mM Hepes, 100 mM KCl and 2 mM MgCl2 (pH 7.4) was layered on the top of PNS and subjected to ultracentrifugation at 182,000 × g for 1.5 h at 4°C. The top layer of the gradient formed after ultracentrifugation will be enriched in LDs. This fraction was resuspended in buffer B and re-centrifuged 270,000 × g for 10 min at 4°C. After this, the top layer, containing the LD-enriched fraction was collected. The quality of the fraction was assessed by performing Western blots for the LD marker, perilipin-2, using an aliquot of the final LD-enriched fraction along with other fractions.

#### Immunofluorescence

MCF7, MDA-MB-231 or T47D cells were plated onto poly-D-lysine-coated eight-well glass chamber slides (20,000–25,000 cells per well) for immunostaining. The cells were fixed with 10% buffered formalin and indirect immunofluorescence was performed. Primary antibodies used were PLIN2 (ab78920 or LSBIO) at 1:500 dilution or LC3B (Cell Signaling Technology) at 1:100 dilution, LAMP2 (novus) at 1:500 dilution, LAMP2a (Abcam125068) at 1:200 dilution. Anti-rabbit Alexa Fluor-594, 647 or 680 or anti- mouse Alexa Fluor-647 (Molecular Probes) was used as the secondary antibody. Lysosomes were detected by lysotracker staining or LAMP2 antibody staining. For Lysotracker deep Red staining, first live cell staining was performed by incubating at 37 0C for 30 min with the dye (dilution:0.2 μL dye in 1 mL media) followed by washing and fixing in formalin and subsequently stained for lipid droplets. Lipid droplets were visualized by Nile Red staining (LD staining kit). DAPI (Vector Labs) was used to stain the nuclei. Cells were visualized with a DM16000 inverted Leica TCS SP5 tandem scanning confocal microscope with a x63/1.40NA oil immersion objective. Images and Z-stacks were produced with three cooled photomultiplier detectors and analyzed with the LAS AF software version 1.6.0 build 1016 (Leica Microsystems, Germany).

Quantification of signals from immunostained cells: For quantifying adiposomes (LD), Leica Confocal SP8 was used to acquire Z-stack images with 63×1.4Na objective. Maximum projection images were analyzed in Definiens Tissue Studio 2.7 for PLIN2 and Nile Red co-registered lipid droplet fluorescence. Frequency of lipid droplets by area were graphed by treatments. For quantification of LDs by spot analysis, uncompressed Tiff images were imported into the Definiens Tissue Studio v4.7 suite (Definiens Inc, Germany). In Tissue Studio the DAPI stain channel was used to segment each cell nucleus based on intensity and size constraints. Next, the Spot stain analysis was applied to the Nile Red stain channel to enumerate lipid droplets within each image. Intensity and size thresholds were used to refine the classification of the lipid spots. The total area of spots and number of nuclei were used to calculate average area of lipid droplets per cell. All thresholds for lipid droplet identification were identical between treatment groups.

#### Tissue microarray and immunohistochemistry

TMA containing formalin-fixed and paraffin-embedded human breast tissue specimens was constructed in Moffitt Cancer Center histology core. The TMA contains 27 normal breast tissues, 30 DCIS, 48 invasive ductal carcinomas without metastasis, 49 invasive ductal carcinomas with metastasis and 48 lymph node macrometastases of breast cancer. 1:100 dilution of PLIN2 antibody (Abcam cat# ab78920) was used for staining. Normal placenta was used as a positive control. For a negative control, an adjacent section of the same tissue was stained without application of primary antibody, and any stain pattern observed was considered as nonspecific binding of the secondary antibody. Immunohistochemical analysis was conducted using digitally scanning slides and scoring by a pathologist. The scoring method used by the pathologist to determine (1) the degree of positivity scored (the positivity of each sample ranged from 0 to 3 and were derived from the product of staining intensity (0–3). A zero score was considered negative, score 1 was weak positive, score 2 was moderate positive, and score 3 was strong positive. (2) The percentage of positive tumors stained. H score was calculated using the following formula H score = 3× (% of cells scored 3)+2×(% of cells scored 2)+1×(% of cells scored 1).

#### Cell growth assessment using imaging

Cells were plated on 96 well plates (Corning Cat. no. 353072) in 200 μL of DMEM supplemented with FBS. Plates were then inserted into the Essen Bioscience Incucyte S3 imaging system with plates imaged every 6 h. After 48 h of growth, cells were treated with inhibitors and pH 7.4/6.5, and imaging continued every 6 h. After seven days from initial plating, Incucyte images were analyzed for confluence using the provided Incucyte S3 software. The confluency was plotted against time, and p value was calculated using the GraphPad Prizm 8.1.1 program. The outer two rows as well as the outer two columns were disregarded from analysis due to edge-related evaporation effects of the plates.

#### siRNA transfections

siRNA specifically targeting TDAG8 as well as a non-targeting control siRNA was purchased from Ambion and siRNAs were transfected using Oligofectamine (Invitrogen Corporation) according to manufacturer’s protocols. All data were graphically represented and statistically analyzed using Microsoft Office Excel 2007 (Microsoft Corporation, Redmond, WA). In all analyses, means and 95% confidence intervals were estimated. Statistical analysis was performed using Student t-test and values were considered significant when the p value was <0.05.

#### qRT-PCR

For real-time PCR, total RNA was isolated using RNeasy miniprep kit (QIAGEN) following the manufacturer’s protocol, followed by first-strand cDNA synthesis using iScript cDNA Synthesis Kit (Bio-Rad) and realtime PCR using SYBR green (Biorad). Data were analyzed by the ΔΔCt method, where gene of interested was normalized to 18 s rRNA or GAPDH. Error bars represent the SD of three independent experiments. Details of sequences of primers used for RT-PCR are given in Table S1.

#### Western blot analysis

Cell lysates were prepared by lysing cells in Nonidet P-40 (Igepal CA-630 or NP-40) lysis buffer with a cocktail of protease and phosphatase inhibitors. 40–80 micrograms of protein per sample were run on polyacrylamide-SDS gels which later were transferred to nitrocellulose membrane and immunoblotted with the indicated antibodies.

#### Oxygen consumption and extracellular acidification measurements

Real-time basal oxygen consumption (OCR) for MCF7-Control and MCF7-OGR1-KO2 cell lines were determined using the Seahorse Extracellular Flux (XF-96) analyzer (Seahorse Bioscience). The XF-96 measures the concentration of oxygen and free protons in the medium above a monolayer of cells in real time. Cells seeded in an XF microplate were cultured for 36 h and subsequently treated with media with pH 7.4 or 6.5 for 48 h. Number of cells in each well was determined using the Celigo imaging cytometer. OCR values were normalized to cell number and plotted as the mean ± SD. In Seahorse assays, on any given day, multiple independent wells (sample replicates) were assayed per group, and each measurement was repeated 3 to 5 times per condition (technical replicates), and the assays were performed independently multiple times (biological replicates). For MCF7 Control and MCF7 OGR1-KO2 cells, there were 5 technical replicates per test and three biological replicates. 1 μM of oligomycin, FCCP and rotenone/antimycin A was used for the assay.

#### Isotope tracing and lipidomics analysis

For ^13^C tracer analysis cells were treated with [UL] ^13^C_6_ Glucose (5 mM), 2-^13^C Acetate (0.5 mM), [UL]^13^C lactate (1 mM) and [UL]^13^C Glutamine (4 mM) as direct metabolic substrates. Tracer was added to the media upon acidification and incubated for 72 h and LDs were isolated for lipidomocs. For 3-^13^C leucine labeling, leucine-free media was substituted with 0.8 mM 3-^13^C Leucine and cells were grown in this culture media for 8 generations. At the time of experiment, the media was replaced with complete label-free media during the 72 h incubation at acid pH and LDs were isolated for lipidomics.

Lipids from LD-enriched fractions were extracted using a modified Folch–extraction approach (Folch et al., 1957). This involves stepwise addition of CHCL3, then MeOH with vigorous vortexing until a final ratio of 4:2:1 CHCl3:MeOH:H2O is obtained. This is then centrifuged and the lower, chloroform layer collected without contamination. An equal volume of CHCl3 is then stepwise added to the remaining aqueous/MeOH layer with vigorous vortexing, followed by centrifugation and harvest of the lower layer, without contamination. This is spun once more, and any remaining upper (aqueous) layer removed. The remaining CHCl3 samples were evaporated under N2 flow. The samples were reconstituted in isopropanol and subsequently subjected to ultra high-pressure liquid chromatography coupled to high resolution mass spectrometry-based global lipidomics analysis, described below.

Extracted lipids were analyzed on a Thermo Scientific Q Exactive orbitrap equipped with a heated electrospray ionization (HESI II) probe in positive and negative ion modes. Separation was achieved on an UHPLC system (Thermo Dionex UltiMate 3000 RS) with Waters BEH C18 column (2.1 × 50 mm, 1.7 μm particle size) maintained at 30°C. Typical injection volumes were 3 μL with a mobile phase flow rate of 500 μL/min. The gradient program consisted of mobile phase A [60:40 acetonitrile/water] and mobile phase B [90:8:2 isopropanol/acetonitrile/water], each containing 10 mM ammonium formate and 0.1% formic acid. The gradient included 32% B at 0 min, 40% B at 1 min, a hold at 40% B until 1.5 min, 45% B at 4 min, 50% B at 5 min, 60% B at 8 min, 70% B at 11 min, and 80% B at 14 min. The total run time is 21 min, including a 3 min equilibration.

Data processing, analysis and interpretation. Lipidomics data file were converted from. raw to .mzxml format using MSConvert a ProteoWizard tool (https://proteowizard.sourceforge.io/download.html) (He et al., 2015). El-MAVEN v0.8.0 was employed for all data processing which involved in feature detection, chromatographic peak alignment, filtering, lipid identification, and data matrix generation as.csv format. Lipids were identified using our internal library based on the El-MAVEN standard parameters includes +/− 5.00 ppm of EIC (extracted ion chromatogram) extraction window, 5 best of the limit number of reported groups per compound, at least 30% peaks above the minimum intensity, 1 peak above minimum quality, minimum signal/blank ratio, and minimum signal/baseline ratios. Polly™ *FirstView* App on Polly El-MAVEN interface were utilized for visualizing relative abundance of ^13^C distribution in lipid droplets of breast cancer cells. Polly™ *PollyPhi* App on Polly El-MAVEN interface were utilized to interpret ^13^C fluxomics data. The natural abundance correction and the fractional enrichment were conducted using labeled LC-MS work flow from Polly Elucidata, where the fractional enrichment analysis depicts the presence of different isotope labels across a lipid species and the natural abundance correction depicts the intensities for each isotopologue after correction with ^12^C. Corna, an open source python-based programming language were used for natural abundance (NA) correction (Raaisa et al., 2020). Briefly, for correcting NA of LCMS data, we generate a correction matrix for each metabolite by calculating mass isotopomer distributions of the labeled tracer and its corresponding indistinguishable isotopes from their known natural abundances. We then multiply inverse of the correction matrix with observed intensities as shown in the following Equation.

$$I_{obs}=CM*I_{corr}$$

$$I_{corr}=CM^{-1}*I_{obs}$$

CM is correction matrix, I_obs_ is observed isotopologue and I_corr_ is corrected isotopologue.

For ^13^C tracer experiments 5 mM [UL] ^13^C_6_ Glucose, 0.5 mM 2-^13^C Acetate, 1 mM [UL]^13^C lactate and 4 mM [UL]^13^C Glutamine were used to treat the cells. For steady state labeling with 3-^13^C leucine, wells were grown in presence of 0.8 mM 3–13C leucine in leucine free media for 8 doublings.

#### NMR sample preparation

Lipid droplets (LDs) from the T47D cancer cell line were dried down overnight by speedvac at room temperature. LDs were re-constituted in non-deuterated chloroform (CHCl3), and supernatant was transferred into a new centrifuge tube and chloroform was evaporated under the nitrogen gas. 1H and 2H NMR analysis of the LDs sample was carried out after dissolving dried lipid sample in 60 μL of CHCl3 (Sigma Aldrich, USA) containing a pyrazine internal standard (4% pyrazine-d4/96% pyrazine).

#### NMR data acquisition and processing

^1^H and ^2^H NMR spectra of LDs were acquired on a 14.1 Tesla Bruker NMR system equipped with 1.7 mm TCI CryoProbe and Avance Neo console (Bruker BioSpin). The operating frequencies to acquire ^1^H and ^2^H NMR spectra were 600 and 92.14 MHz, respectively. For ^1^H NMR acquisition, a relaxation delay (d1) of 2 s, acquisition time of 2 s and 13,157 data points were digitized over a spectral width (sw) of 11 ppm. The deuterium lock channel was used to record ^2^H NMR data. Acquisition time of 2 s, relaxation delay of 2 s, flip angle of 90°, 2048 number of scans, acquired size (TD) of 2173 and spectral width of 11 ppm were used during the acquisition. All NMR spectra were acquired at 25°C. MestReNova (v14.0.1-23284, S.L, USA) software was used to process all of the NMR data. ^1^H NMR were Fourier transformed with an exponential line-broadening factor of 0.5 Hz and zero filling to 64 k data points followed by manual phase and automatic spline baseline correction. ^2^H NMR spectra were processed with 1.00 Hz exponential line broadening, zero filled to 8 k data points, manually phased and automatic splines baseline correction was applied. The peak fitting tool was used to extract the peak areas of fatty acid signals in ^1^H and ^2^H NMR spectra.

#### Chemical shift assignment and *de novo* lipogenesis (DNL) in LDs

Proton and deuterium chemical shift assignments of the fatty acyl chain functional moieties in ^1^H and ^2^H NMR spectra were determined by using the values previously described (Duarte et al., 2014) (Figure 2D). The percentage of lipid synthesized by DNL during D_2_O exposure was determined by the ratio of methyl ^2^H enrichment in the lipid pool to the water ^2^H enrichment in the cell culture (i.e. 25%). The lipid methyl ^2^H enrichment was calculated using the extracted peak areas of the terminal methyl of fats and pyrazine in the ^1^H spectrum, and the deuterated terminal methyl and pyrazine-d_4_ from the ^2^H NMR spectra. The stoichiometry of the deuterium to proton signals of methyl was compared with the pyrazine standard (Duarte et al., 2014).

#### Clinical data analysis

Gene expression and survival analyses of acid sensors in patients with breast carcinoma were analyzed from The Cancer Genome Atlas (TCGA) database (http://cancergenome.nih.gov), GEPIA (Gene Expression Profiling Interactive Analysis) http://gepia.cancer-pku.cn/and ULCAN portal (http://ualcan.path.uab.edu/).

#### Generation of CRISPR knock out cell lines

Specific gRNAs targeting TDAG8 or OGR1 were designed (using http://crispr.mit.edu/) and cloned into lentiCRISPR V2 construct (Feng Zhang lab) using established protocols (Sanjana et al., 2014). Lentiviral particles were produced by transiently transfecting 293-FT cells with lentiCRISPR V2 and lentiviral packaging constructs (pLP1, pLP2, VSVG; ViraPower Lentiviral Expression System, Invitrogen) using Fugene HD (Roche). Culture supernatants containing lentivirus were collected 48 and 72 h after transfection, pooled after removing cell debris by centrifugation at 1500 rpm for 5 min and concentrated using Lenti-X Concentrator (clonetech) and stored at −80°C. Cells were infected using the virus in polybrene (6 μg/mL) containing media. MCF7, T47D and MDA-MB-231 cells were transduced with lentiviral particles for 24 h and subsequently subjected to antibiotic selection (puromycin 2 μg/mL) for generating single cell derived clones. For checking the presence of the desired edits, genomic DNA was isolated, targeted region in the genome was amplified and sequenced. Two gRNAs (one at the 5′ end and the other at the 3′ end of the gene) were used to edit out the entire gene (Figure S5C). In this case, diagnostic PCRs were carried out to amplify the gene by using primers flanking 5′ and −3′ end of the gene. Additionally, real time PCR was carried out to assess the mRNA levels in clones where the entire gene was edited out using two gRNAs. The OGR1 knockout cell line generated using two sgRNA is named as OGR1KO2 for MCF7. Both KO1 and KO2 T47D cell lines are generated by OGR1 exon2 deletion as described in Figure S5C. As control gRNA, a sequence targeting EGFP that do not target any mammalian genomic sequence was used. Alternatively, gRNA targeting the 5′ region of OGR1 exon 2 alone was also used to edit the protein coding sequences. The sequences of primers used for cloning OGR1, TDAG8 and EGFP gRNAs are given in the Key resources table.

#### Ectopic expression of OGR1

For generating stable cell lines expressing OGR1, MCF7 cells were transfected with OGR1(Myc-DDK) tag (Origene, CAT#: RC229950) or empty vector control (pCMV6-Entry Mammalian Expression Vector, Origene,CAT#:PS100001) using Fugene HD. Stable clones were selected using geneticin (neomycin sulphate), and OGR1 expression was confirmed using RT-PCR and Western blot analysis. In rescue experiments, OGR1 CRISPR knockout cells were transiently transfected with the above-mentioned vectors and subsequently induced OGR1 signaling using culture media with different pH for 48 h and assessed LD accumulation or expression of lipogenic enzymes. For pAKT western blots, cells were induced with pH (74 or 6.5) media for 1 h as pAKT induction was higher at that time point.

#### IP one assay

To confirm if OGR1 activation leads to Gq coupling, PLC activation and subsequent production of IP3, we measured IP1 (myo-Inositol 1-Phosphate) using IP-one assay (IP-one -Gq HTRT kit, Cisbio) as IP1 can be used as a surrogate for IP3. For this, MCF7 cells stably expressing OGR1 and OGR1 depleted MCF7 (OGR1-KO) were plated in 384 well plates and allowed to grow for 24 h. Cells were then pretreated with media at different pH with or without inhibitors (Edelfosine or YM compound) for 18 h. Assay was performed according to manufacturer’s protocols and the plate was read using Envision Plate Reader. The time resolved-fluorescence resonance energy transfer (TR-FRET) 665 nm/620 nm ratio, which is inversely proportional to the IP1 accumulation, was used to measure the amount of IP1 produced. Using the standard curve generated by known concentrations of IP1, the fluorescence ratios from the treated samples were converted to IP1 concentration. Assay was repeated at least 3 times with a minimum of 3 replicates. Data were analyzed using the GraphPad Prism software.

#### Triglyceride measurements

Triglycerides (TG) from cell lines as well as tumor tissues were measured using Triglyceride-Glo Assay (Promega). In this assay, TG levels are determined by measuring Glycerol that is released from Triglycerides in an enzymatic reaction with a lipase. One molecule of glycerol per molecule if TG is released by this reaction. Glycerol is then measured in a coupled reaction that links the production of NADH to activation of proluciferin that produces light by luciferase activity. The amount of TG is determined from difference in glycerol measured in absence (free glycerol) and presence (total glycerol) of lipase. TG assay was performed according to manufacturer’s protocols. MCF7, T47D and MDA-MB-231 cells were seeded in 96 well plates, subsequently treated with different pH media with or without inhibitors. 48 h after treatments, cell was lysed with or without lipase and released glycerol was measured using detection reagent. Luminescence was read using the Flexstation3 Multimode Plate Reader (Molecular Devices). For measuring TGs from tumor tissues, 10 mg of cryopreserved tumor tissue was lysed in 250 μL of glycerol lysis buffer and diluted with lysis butter with or without lipase. TG levels were determined as the difference between total (with lipase) and free glycerol (without lipase).

#### Transmission electron microscopy

Cells grown in culture plates treated with pH 7.4 or 6.5 media for 48 h were processed using standard protocols for TEM (Graham and Orenstein, 2007). Briefly, cells were fixed in 2.5% glutaraldehyde in 0.1 M sodium cacodylate buffer, pH 7.4 for 45 min. Samples were rinsed three times in sodium cacodylate rinse buffer and subjected to osmication by exposing cells in 1% reduced osmium (equal volumes of 2% aqueous osmium tetroxide and 3% aqueous potassium ferrocyanide) for 2 h at 4°C, followed by several rinses in buffer. Cells were collected by gentle scraping in fresh buffer and pelleted by centrifugation. Cell pellets were then exposed to a second osmication step in 1% aqueous osmium for 30 min at room temperature, followed by centrifugation to remove osmium. Samples were washed in buffer (4 × 5minutes) followed by dehydration in graded alcohols (35%,50%,70% 90%, 100% ethanol). Further, infiltration into epoxy resin (Embed812) consisted in two changes in 100% acetone (2 × 15 min), incubation in equal volume mixture of acetone and resin and 2:1 resin to acetone for 1 h each at room temperature. This was followed by two changes into pure fresh resin for 2 h each at room temperature. The final change into resin was incubated for 3 h and cells pelleted by centrifugation at 3000 g for 10 min, before polymerizing resin in 65°C oven for 48 h. Cured blocks were trimmed and 100 nm sections cut on a Reichert Ultracut E microtome and placed on 200 mesh copper grids. No contrast staining was used prior to image capture done on a JEOL 1400 TEM equipped with a Gatan Orius digital camera.

### QUANTIFICATION AND STATISTICAL ANALYSIS

Data ware analyzed using GraphPad Prism 8 and represented as mean ± S.D. Data were compared using unpaired t test, one-way ANOVA or two-way ANOVA, as indicated in the figure legends. p values of <0.05 were considered significant. Information on biological replicates (N) and significance (p values) of individual tests can be found in figure legends.

## Supplementary Material

1

## Figures and Tables

**Table:** KEY RESOURCES TABLE

REAGENT or RESOURCE	SOURCE	IDENTIFIER
Antibodies		
Rabbit polyclonal OGR1	Abcam	ab72500,; RRID:AB_1523738
Rabbit polyclonal OGR1	LSBio	LS-A1194; RRID:AB_591626
Rabbit polyclonal PLIN2/ADFP	Abcam	ab78920; RRID:AB_2040415
Rabbit monoclonal LC3B	CST	3868S; RRID:AB_2137707
Rabbit monoclonal phospho AKT S473	CST	4060; RRID:AB_2315049
Rabbit polyclonal phospho AKT T308	CST	9275; RRID:AB_329828
Rabbit polyclonal Total AKT	CST	9272; RRID:AB_329827
Rabbit monoclonal GAPDH	Abcam	Ab181602;RRID:AB_2630358
Mouse monoclonal Actin	Sigma	#A1978; RRID:AB_476692
Rabbit polyclonal PLIN2	LSBio	B4850; RRID:AB_10801440
LAMP2/CD107b	Novus	NBP2-22217; RRID:AB_2722697
LAMP2a/Lysosome marker	Abcam	125,068; RRID:AB_10971511
Goat anti-Mouse IgG (H+L) Alexafluor 647	Molecular Probes	A32728; RRID:AB_2633277
Goat anti Rabbit Alexafluor 594	Molecular Probes	A11012; RRID:AB_2534079
Goat anti-Rabbit Alexafluor 647	Molecular Probes	A21245; RRID:AB_2535813
Goat anti-Rabbit Alexafluor 680	Molecular Probes	A27042; RRID:AB_2536103
Rabbit polyclonal phospho NFkB	CST	3033/93H1; RRID:AB_331284
Total NFkB	CST	D14E12; RRID:AB_2799570
Na/K+ATPase	Abcam	ab76020; RRID:AB_1310695
Rabbit monoclonal Fatty acid Synthase	CST	3180; RRID:AB_2100796
Rabbit monoclonal ACSS2/AceCS1	CST	3658; RRID:AB_2222710
Rabbit polyclonal ACLY	CST	4332; RRID:AB_2223744
Rabbit monoclonal TFEB	CST	37785;RRID:AB_2799119
Rabbit monoclonal ACC	CST	3676; RRID:AB_2219397
Rabbit monoclonal PERK (D11A8)	CST	5683; RRID:AB_10841299
Rabbit polyclonal Phospho IRE1α (S724)	Abcam	ab124945; RRID:AB_11001365
Donkey Anti-Rabbit IgG Peroxidase conjugated	Pierce	PI-31458; RRID:AB_228213
Donkey Anti-Mouse IgG Peroxidase conjugated	Pierce	PI-31450; RRID:AB_2534691
Bacterial and virus strains		
lentiCRISPR V2	Addgene	52961
Stbl3 competent E. coli cells	Invitrogen	C737303
DH5α Competent E. coli cells	Invitrogen	18258012
Biological samples		
Breast Tissue Micro Array	Moffitt Cancer Center	N/A
Chemicals, peptides, and recombinant proteins		
TOFA	Cayman Chemicals	10005263
C75	Cayman Chemicals	191282-48-1
Etomoxir	Cayman Chemicals	11969
GDC0941	Selleck Chemicals	Catalog No. S1065
MK2206	Selleck Chemicals	Catalog No. S1078
Chloroquine diphosphate	Sigma	C6628
Bafilomycin A1	Cayman Chemicals	Item No.11038
PLC inhibitor U-73122 hydrate	Sigma	U6756
Edelfosine	Tocris	Cat.No.3022
Ogerin	Sigma	SML1482
DMEM, High Glucose	Gibco	11965-092
DMEM w/o L-glutamine, leucine, sodium pyruvate	MP Biomedicals	091642149
DMEM, no glucose	Gibco	11966-025
DMEM without Leucine	CRYSTALGEN INC	NC0565703
PIPES	Sigma	P1851
HEPES	SIGMA	H4034
Delipidated serum	Gemini Bioproducts	900-123
^13^C5 L-GLUTAMINE	Cambridge Isotope	1822-H-0.1
D-GLUCOSE (U-^13^C6, 99%)	Cambridge Isotope	CLM-1396-5
L-Leucine 3-^13^C	Sigma	604828
SODIUM ACETATE(2-^13^C)	Cambridge Isotope Lab	CLM-381-5
SODIUM L-LACTATE-^13^C3 SOLUTION	Sigma	485926
Fugene	Promega	PR-E2311
SYBR Green (Sso Advanced)	Biorad	172-5271
iScript cDNA synthesis Kit	Biorad	1708891
Power SYBR green	ABI	4368702
MTT	Sigma	M2128
Oligofectamin	Invitrogen	12252-011
Lipofectamine RNAiMAX	Invitrogen	13778075
Thiazolyl Blue MTT	Sigma	M2128-500MG
Mounting medium with DAPI	Vector Labs	H-1200
Glass chamber slides	LabTek	12-565-8
Normal Goat Serum	Fisher	PI-31873
Tricine	Sigma	T0377
Lysotracker Deep Red	Invitrogen	L12492
Critical commercial assays		
QIAamp DNA Mini Kit	Qiagen	51304
RNeasy Plus Mini Kit	Qiagen	74134
LD Fluorescence Assay Kit	Cayman Chemicals	500001
iScript cDNA synthesis Kit	Biorad	1708891
SYBR Green (Sso Advanced)	Biorad	172-5271
Deposited data		
Lipidomics Data	This paper, https://zenodo.org/record/6449175	Zenodo Data: https://doi.org/10.5281/zenodo.6449175
Experimental models: Cell lines		
Human: MCF7	ATCC/PSON	N/A
Human: T47D	ATCC/PSON	N/A
Human: MDA-MB-231	Robert Gillies’s Lab, Moffitt Cancer Center	N/A
Experimental models: Organisms/strains		
Mouse: 6-8 weeks old Female NSG	Jackson Labs	N/A
Oligonucleotides		
EGFP-gRNA-OligoF-5′ CACCG GGGCGAGGAGCTGTTCACCG	This paper	N/A
EGFP-gRNA-OligoR -5′ AAAC CGGTGAACAGCTCCTCGCCCC	This paper	N/A
OGR1-gRNA-OligoF1-5′ CACCG GGTGGTCTATGTTACCGTGC	This paper	N/A
OGR1-gRNA-OligoR1-5′ AAACGC ACGGTAACATAGACCACCC	This paper	N/A
OGR13′gRNA-OligoF-CACCGT CACGTGGAGCCACCCGCGG	This paper	N/A
OGR13′gRNA-R-AAACCCG CGGGTGGCTCCACGTGAC	This paper	N/A
TDAG8-gRNA-OligoF1-CACCGG TCAGCATTCCAGCCAATAT	This paper	N/A
TDAG8-gRNA-OligoR1-AAAC ATATTGGCTGGAATGCTGACC	This paper	N/A
TDAG8-3′ gRNA-OligoF-CACCGTCATTTAATAAAACGCAGGG	This paper	N/A
TDAG83′gRNA-OligoR- AAAC CCCTGCGTTTTATTAAATGAC	This paper	N/A
Recombinant DNA		
lentiCRISPR V2	Addgene	52961
lentiCRISPR constructs with gRNA inserts listed above	This paper	N/A
OGR1(Myc-DDK) tag	Origene	CAT#: RC229950
pCMV6-Entry	Origene	CAT#: PS100001
Software and algorithms		
Polly El-MAVEN	El-MAVEN v0.8.0	N/A
MSConvert	He et al., 2015	https://proteowizard.sourceforge.io/download.html
LAS AF software version 1.6.0 build 1016	Leica Microsystems, Germany	N/A
Definiens Tissue Studio v4.7 suite	Definiens Inc, Germany	N/A
Incucyte S3	Select Science	N/A
Other		
GPR68/OGR1siRNA	Ambion	Cat. #4427037
TDAG8/GPR65siRNA	Ambion	s16100
Silencer Negative Control siRNA	Ambion	AM4611

## Highlights

- Extracellular acidosis induces robust accumulation of cytoplasmic lipid droplets
- This adaptive mechanism is triggered by the acid-sensing GPCR OGR1 (GPR68)
- Lipid droplets originate from autophagic breakdown of ketogenic amino acids
- Inhibition of lipogenic phenotype is a targetable metabolic vulnerability
