# Supplementary material for: Lipogenesis mediated by OGR1 regulates metabolic adaptation to acid stress in cancer cells via autophagy
Source: Cell Rep. Author manuscript; Available in PMC 2022 May 27. (PMC9137419; doi:10.1016/j.celrep.2022.110796)
Supplement: 1 [file NIHMS1806833-supplement-1.pdf]

Cell Reports, Volume *39*

Supplemental information

Lipogenesis mediated by OGR1 regulates metabolic adaptation to acid stress in cancer cells via autophagy

Smitha Pillai, Iqbal Mahmud, Rohit Mahar, Crystal Griffith, Michael Langsen, Jonathan Nguyen, Jonathan W. Wojtkowiak, Pawel Swietach, Robert A. Gatenby, Marilyn M. Bui, Matthew E. Merritt, Patricia McDonald, Timothy J. Garrett, and Robert J. Gillies

Supplemental Information

Table S1: **Primer sequences used in Real-time qPCR assays**. Related to STAR Methods.

Gene	Forward primer	Reverse Primer
OGR1	5′-ACTTCGGCTACCTGCAGATC-3′	5′-AGCCCACGCTGATGTAGATG-3′
TDAG8	5′-AAG GAA AGT GAA CTA GGA ATT TAC CTC TTC-3′	5′-GAA AGT CCA GTT GTC TTT ATT CCA GGT ATA ATC-3′
18SrRNA	5'-CTC AAC ACG GGA AAC CTC AC-3'	5'-AAA TCG CTC CAC CAA CTA AGA A-3'
GAPDH	5′-GGTGGTCTCCTCTGACTTCAAGA-3′	5′-GTTGCTGTAGCCAAATTCGTTGT-3'

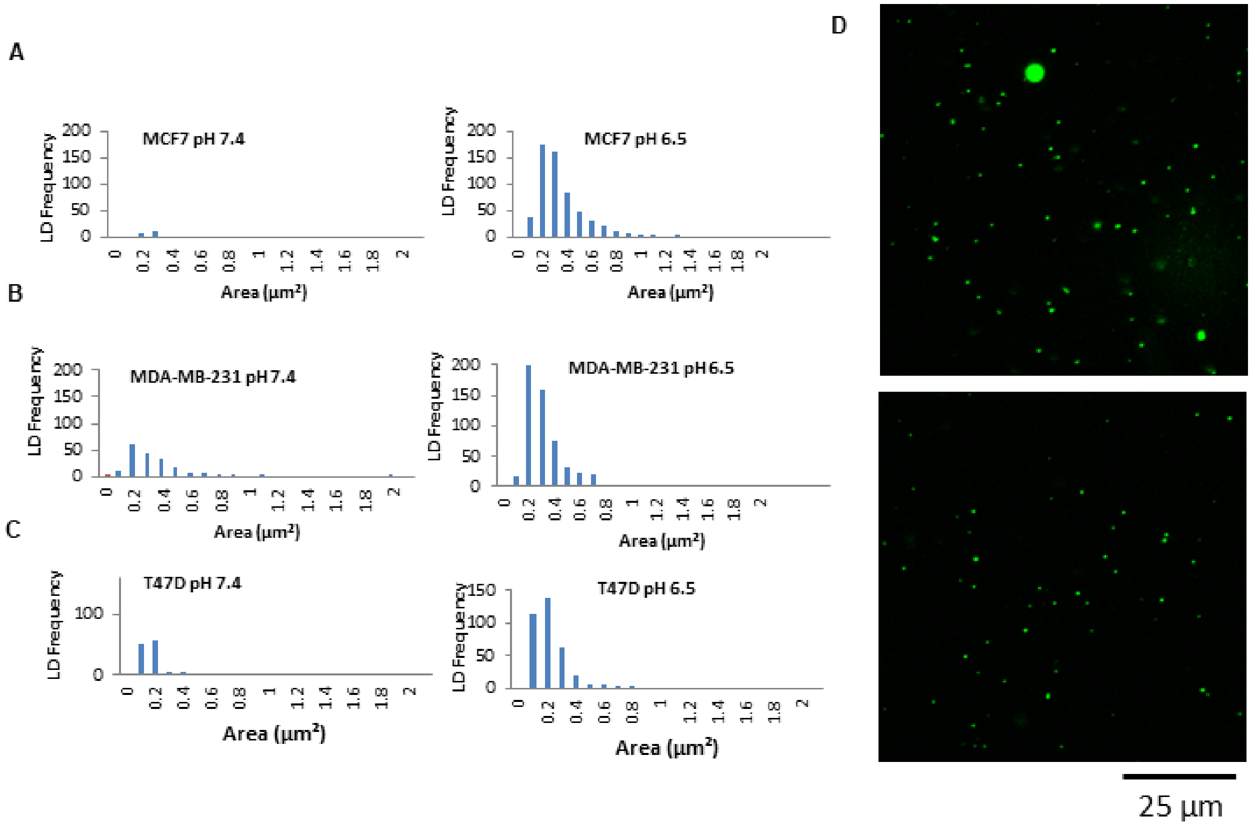

Figure S1

Size distribution of lipid droplets. Related to Figure 1. (A-C) Representative histograms for the distribution of LDs in cells grown in normal pH (7.4) and acidic pH (6.5). Three biological replicated were performed. (D) LDs isolated from MCF7 cells grown in acidic media (pH6.5) stained with Nile Red as seen by confocal microscopy.

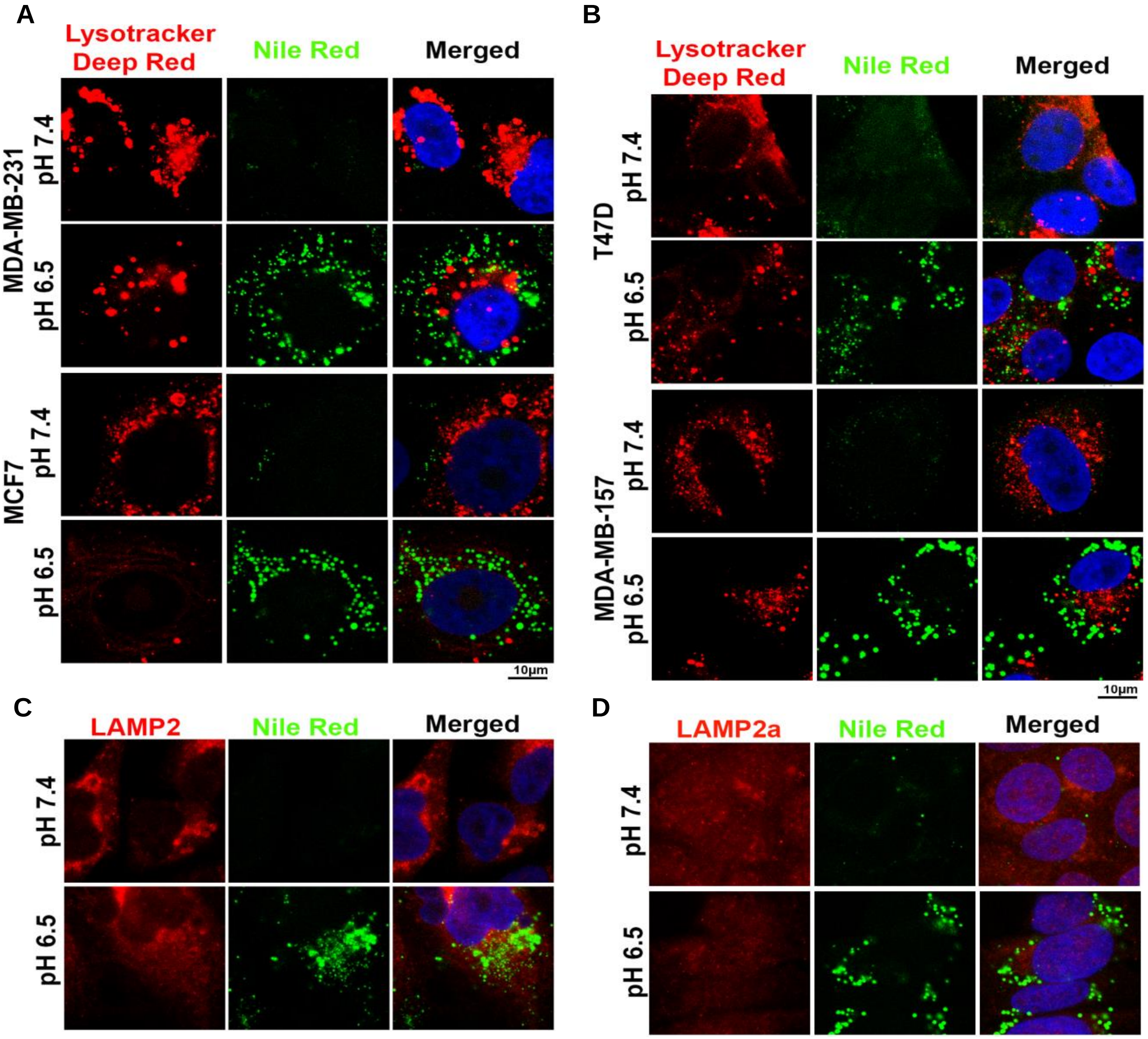

Figure S2

Nile Red does not stain lysosomes in breast cancer cell lines. Related to Figure 1. (A) To confirm that nile red stains lipid droplets and not lysosomes, MDA-MB-231 and MCF7 cells treated with pH 7.4 or 6.5 media were stained with Lysotracker Deep Red (red). After washing and fixing the cells in formalin, neutral lipids were stained with Nile Red (green). Both of these fluorescent probes stained different cellular organelles or compartments. (B) similar staining patterns were observed inT47D and MDA-MB-157 cells. Lysotracker Deep Red (Red), Nile Red (green), DAPI (Blue). (C &D) Nile Red and Lysosome marker antibodies do not stain the same organelles. (C) MDA-MB-231 cells fixed with formalin were stained with LAMP2 antibody (novus(H4B4) [NBP2-22217) followed by Nile Red. (D) MCF7 cells stained with LAMP2a, lysosome marker (ab125068, Abcam). LAMP2 (Red), Nile Red (Green), DAPI (Blue).

A

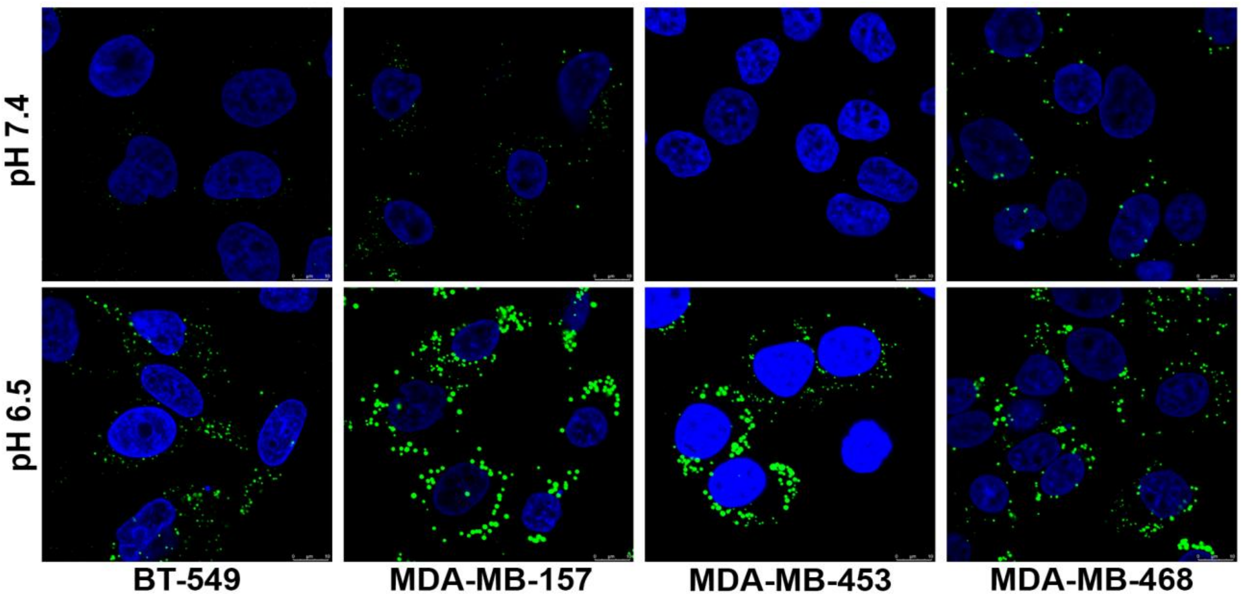

B

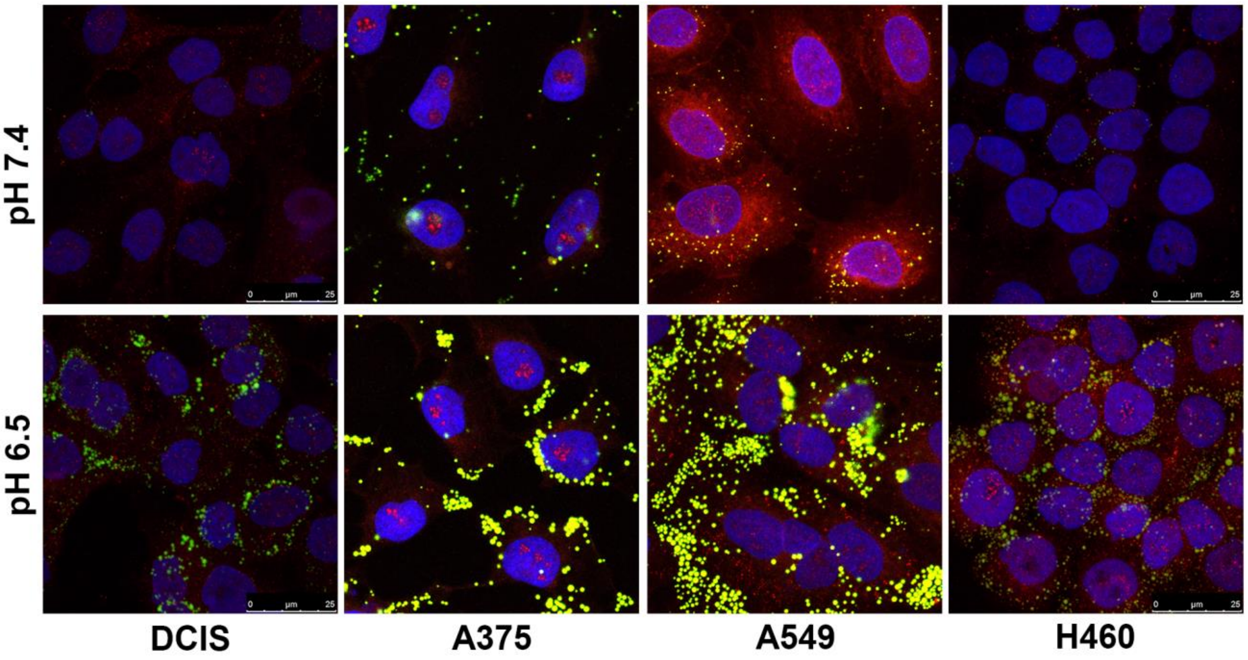

Figure S3

Acidic pH induces LD accumulation in cancer cells. Related to Figure 1. (A&B) Triple negative breast cancer (TNBC) cell lines BT-549, MDA-MB-157, MDA-MB-453, MDA-MB-468 as well as DCIS, A375 (melanoma) and lung cancer cell lines A549 and H460 accumulated LDs when treated with acidic media (pH 6.5) compared to neutral media (pH7.4). Nile Red (Green), DAPI (Blue) in A. PLIN2 (Red) Nile Red (Green), DAPI (Blue) in B.

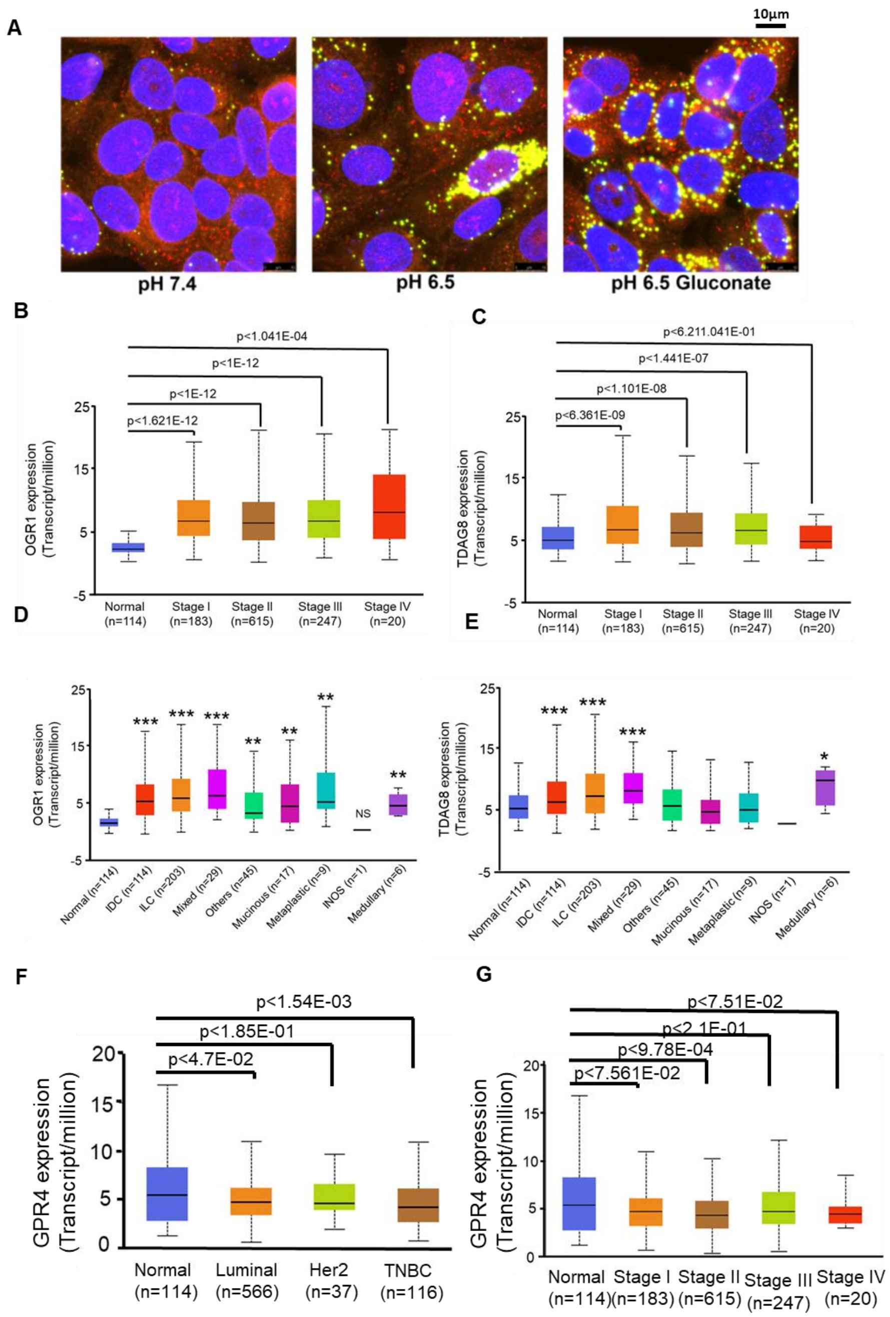

Figure S4

Correlation of acid-sensing receptor expression and breast cancer progression. Related to Figure 3. (A) MCF7 cells treated with gluconate media with pH 6.5 to maintain low pHe and high pHi showed accumulation of LDs. PLIN2 (Red) Nile Red (Green), DAPI (Blue). (B&C) OGR1 and TDAG8 expression are correlated with breast cancer disease progression. OGR1 is overexpressed in breast tumors (stage I-IV) compared to the normal breast tissue. TDAG8 expression is elevated in stages I-III compared to normal breast tissue. Data source: TCGA. (D&E) OGR1 and TDAG8 are overexpressed in different histological subtypes of breast tumors compared to normal breast tissue as seen from TCGA database. (F&G) GPR4 was not overexpressed in breast tumors compared to normal breast tissue and was not correlated with disease progression.

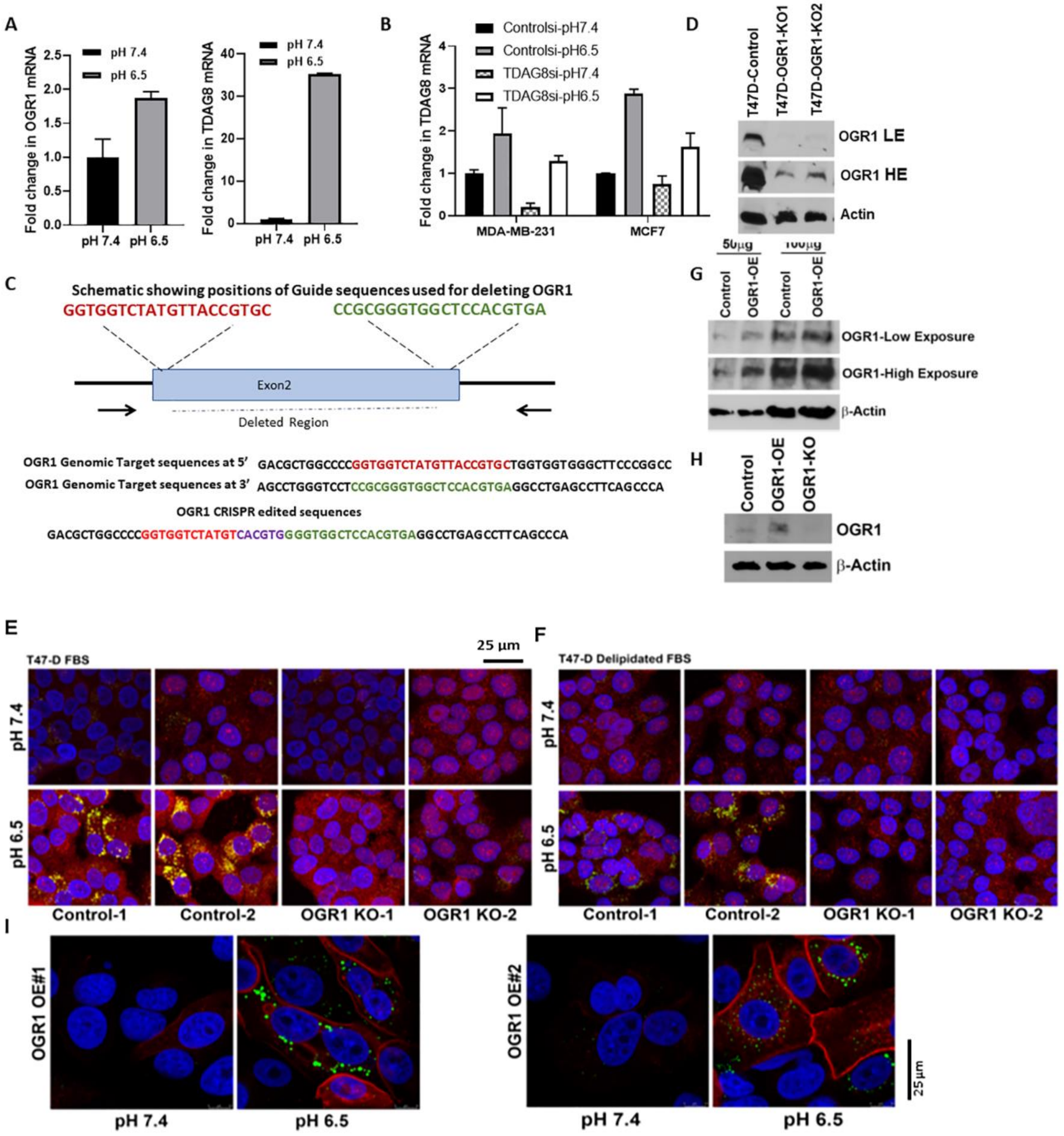

Figure S5

Acid Receptors are induced by low pH in MCF7 cells. Related to Figure 3, 4, 5 and STAR Methods. (A). MCF7 cells when grown in low pH media for 72 hours shows increase in expression of OGR1 and TDAG8 mRNA as seen by qPCR. (B) Depletion of TDAG8 by siRNA transfection was not effective as low pH treatment induced expression of TDAG8 in both MCF7 and MDA-MB-231 cells. (C) Schematic showing positions of Guide sequences used for deleting OGR1. gRNA targeting 5' and 3' of OGR1 coding sequences (exon 2) were used to edit out OGR1. Arrows represent primers designed upstream (forward primers) and downstream (reverse primer) to PCR amplify the edited region. Sequencing of the PCR product revealed the deletion of OGR1 coding sequences. (D-F) OGR1 knockdown abrogates LD accumulation in T47D cells grown in acidic media with FBS or with delipidated

FBS as seen by confocal microscopy (A&B). Perilipin 2 (PLIN2, red), Nile Red (green), DAPI (blue). (D) Western blot analysis showing depletion of OGR1 in T47D OGR1 knockout cells. LE is low exposure. (G&H) Validation of OGR1 antibody for Western blot analysis. (G) Increasing concentrations of lysates from MCF7 cells ectopically expressing OGR1 or controls (pCMV empty vector transfected) show increasing levels of a specific band with expected molecular weight (~40kDa) of OGR1 as seen by western blot analysis using OGR1 antibody (Cat. Number LS-A1194, LSBio). (F) OGR1 specific band is absent in cells depleted of OGR1 by CRISPR/Cas9. (I) MCF7 cells ectopically expressing OGR1 show enhanced expression of the receptor on cell surface as well as LD accumulation at pH 6.5. MCF7 cells were transfected with pCMV-OGR1 with Myc Tag and stably expressing clones were selected. Myc-tag (Red), Nile Red (Green), DAPI (Blue).

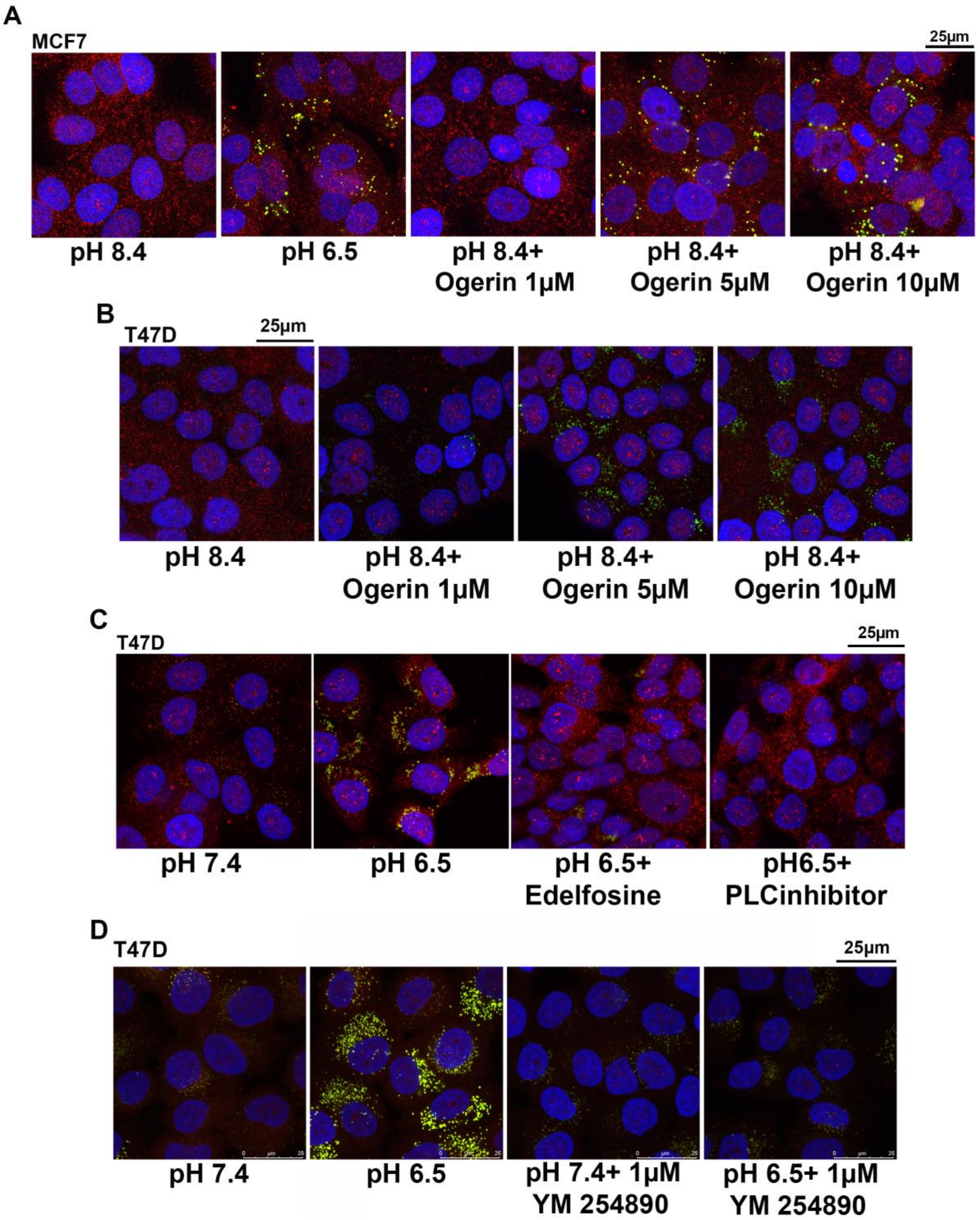

Figure S6

OGR1 agonist Ogerin induces **LD formation and inhibition of signaling downstream of OGR1 abrogates LD accumulation**. Related to Figure 4. (A&B) MCF7 and T47D cells treated with Ogerin, a positive allosteric modulator of OGR1, induced LD accumulation at pH8.4. Perilipin 2 (PLIN2, red), Nile Red (green), DAPI (blue). (C) LD accumulation is inhibited by pharmacological inhibitors of PLC, Edelfosine (5μM) or U73122 (2.5 μM) inhibits accumulation of LDs under low pH. Perilipin 2 (PLIN2, red), Nile Red (green), DAPI (blue). (D) T47D cells treated with YM254890, a cyclic peptide inhibitor of Gq/11 coupled GPCR signaling affects LD accumulation.

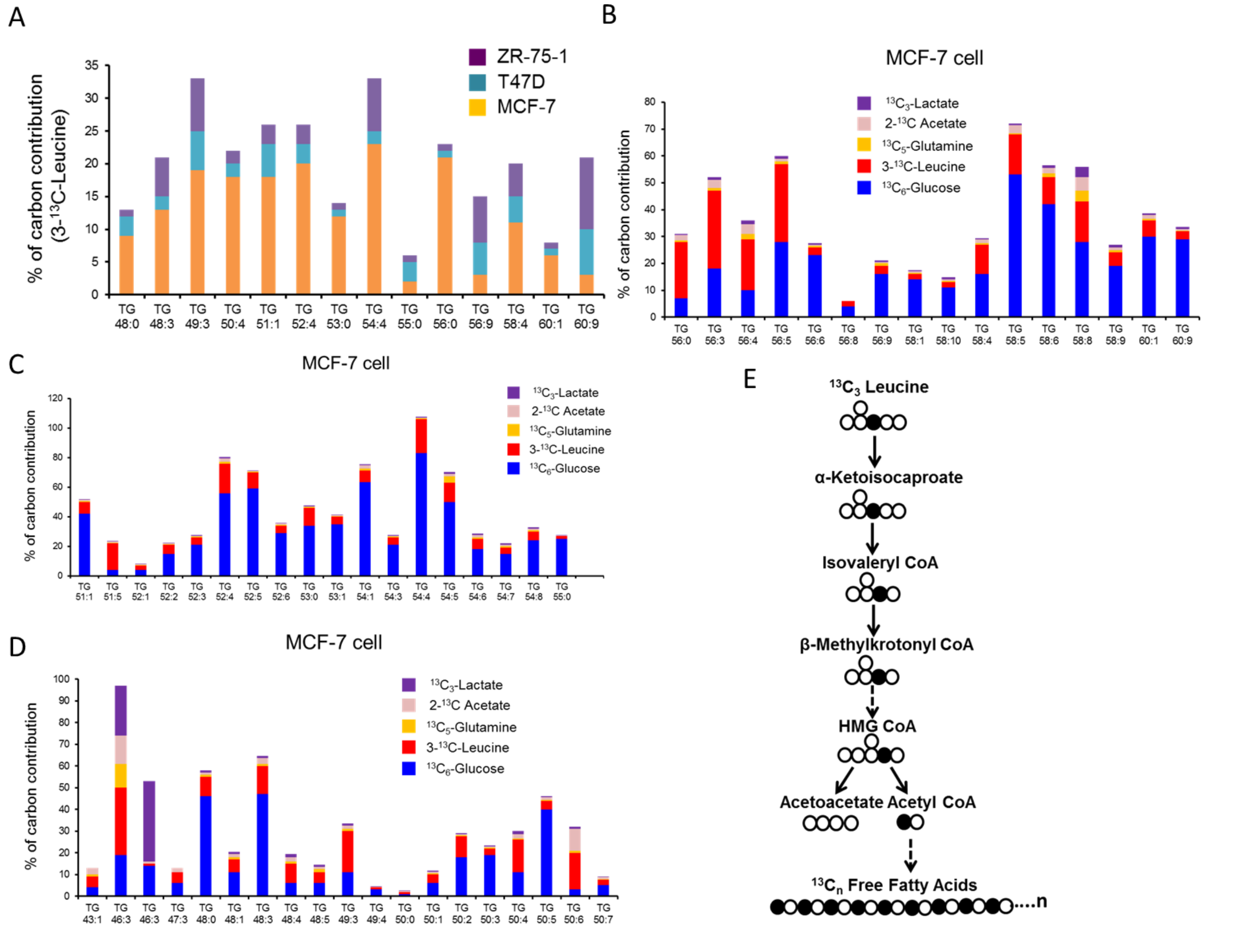

Figure S7

Comparative labeling activity of ^{13}C-labeled glucose, lactate, glutamine, acetate, and leucine in TGs isolaled from LDs. Related to Figure 6. (A) Estimated percentage of carbon contribution to LD-derived TGs isolated from different breast cancer cell lines labeled using ^{13}C-labeled leucine. Cells were labeled by growing 8 generations in media supplemented with ^{13}C-labeled leucine and LDs were induced in label free acidic media via autophagy. (B-D) Percent of carbon contribution to LD-derived TGs isolated from MCF-7 cells by different carbon sources (^{13}C-labeled glucose, lactate, glutamine, and acetate supplemented in cell culture media, and leucine (mobilized from labeled intracellular proteins via acid induced autophagy). (E) Schematic showing flow of carbon during leucine catabolism from 3-^{13}C labeled leucine towards lipid production in LDs.
